# The synergistic interaction of MEK and PI3K inhibitors is modulated by mTOR inhibition

**DOI:** 10.1038/bjc.2012.70

**Published:** 2012-03-13

**Authors:** E J Haagensen, S Kyle, G S Beale, R J Maxwell, D R Newell

**Affiliations:** 1 Drug Discovery and Imaging, Newcastle Cancer Centre, Northern Institute for Cancer Research, Medical School, Newcastle University, Paul O’Gorman Building, Framlington Place, Newcastle-upon-Tyne NE2 4HH, UK

**Keywords:** PI3K, MEK, combination, mTOR, colorectal

## Abstract

**Background:**

Combined targeting of MAPK and PI3K signalling pathways may be necessary for optimal therapeutic activity in cancer. This study evaluated the MEK inhibitors AZD6244 and PD0325901, alone and in combination with the dual mTOR/PI3K inhibitor NVP-BEZ235 or the PI3K inhibitor GDC-0941, in three colorectal cancer cell lines.

**Methods:**

Growth inhibition, survival and signal transduction were measured using the Sulforhodamine B assay, clonogenicity and western blotting, respectively, in HCT116, HT29 and DLD1 cell lines.

**Results:**

All MEK/PI3K inhibitor combinations exhibited marked synergistic growth inhibition; however, GDC-0941 displayed greater synergy in combination with either MEK inhibitor. NVP-BEZ235 exhibited stronger inhibition of 4EBP1 phosphorylation, and similar inhibition of S6 and AKT phosphorylation, compared with GDC-0941. Both PD0325901 and AZD6244 inhibited ERK phosphorylation, and with MEK/PI3K inhibitor combinations inhibition of S6 phosphorylation was increased. The reduced synergy exhibited by NVP-BEZ235 in combination with MEK inhibitors, compared with GDC-0941, may be due to inhibition of mTOR, and the addition of the mTORC1/2 inhibitor KU0063794 compromised the synergy of GDC-0941:PD0325901 combinations.

**Conclusion:**

These studies confirm that dual targeting of PI3K and MEK can induce synergistic growth inhibition; however, the combination of specific PI3K inhibitors, rather than dual mTOR/PI3K inhibitors, with MEK inhibitors results in greater synergy.

Targeted anticancer treatments are an important and evolving new approach to cancer therapy and, in order to treat tumours with defects in multiple oncogenic pathways, combinations of targeted agents are being actively investigated.

In the MAPK pathway, which is commonly activated in human cancers, receptor tyrosine kinase (RTK) activation causes phosphorylation of Ras *via* adaptor proteins. Ras then activates the Raf-MEK-ERK kinase cascade, and ERK phosphorylation leads to the activation of >100 downstream substrates involved in a myriad of cellular processes such as proliferation, survival, transformation, translational control and cytoskeletal rearrangements. This pathway can become constitutively activated by overexpression or mutation of RTKs, and mutations of Ras, especially the KRas isoform (Bos, 1989), and Raf, typically in BRaf at V600E (Davies *et al*, 2002). Hence, small molecule inhibitors targeted against this pathway have been developed. Allosteric inhibitors of MEK are particularly promising as they exhibit marked selectivity for MEK over other protein kinases. The cellular effects of MEK inhibitors are primarily cytostatic and thus reversible (Kohno and Pouyssegur, 2006). The benzimidazole derivative AZD6244 (ARRY-142886, Selumetinib) has exhibited promising selective anticancer efficacy *in vitro* and *in vivo* (Davies *et al*, 2007; Yeh *et al*, 2007), and was well tolerated in phase II clinical trials (Bodoky *et al*, 2011). In contrast, PD0325901, a structural analogue of CI-1040 with improved pharmacological and biopharmaceutical properties (Barrett *et al*, 2008), was associated with adverse effects, such as retinal vein occlusion (Haura *et al*, 2010), despite promising *in-vitro* and *in-vivo* preclinical activity (Liu and Xing, 2008; Hennig *et al*, 2010).

The PI3K/AKT pathway also has a central role in a range of cellular responses such as survival, proliferation, metabolism and growth, and is commonly deregulated in human cancers. Receptor tyrosine kinase activation causes phosphorylation of PI3K *via* adaptor proteins, and PI3K then phosphorylates PIP_2_ to PIP_3_, causing AKT activation *via* two crucial phosphorylation events at threonine 308 catalysed by PDK1 and at serine 473, which may be catalysed by mTORC2 (Sarbassov *et al*, 2005). AKT recognises and phosphorylates many cytoplasmic and nuclear substrates to control numerous crucial cellular functions. Genetic aberrations of PI3K pathway components, such as PTEN loss, PI3K amplification/mutation, AKT mutation or RTK activation, cause constitutive signalling of the PI3K-AKT pathway leading to tumourigenesis (Yuan and Cantley, 2008); hence, PI3K pathway inhibitors have been developed. The dual pan PI3K/mTOR inhibitor, NVP-BEZ235, inhibits cellular proliferation both *in vitro* and *in vivo* and is currently undergoing phase I/II clinical trials (Maira *et al*, 2008; Serra *et al*, 2008; Baumann *et al*, 2009). The thienopyrimidine PI3K inhibitor, GDC-0941, preferentially inhibits the *α* and *δ* p110 isoforms of PI3K over the *β* and *γ* isoforms in an ATP-competitive manner, has potent preclinical tumour growth inhibitory activity, and has recently entered phase I trials (Folkes *et al*, 2008; Maira *et al*, 2009; Raynaud *et al*, 2009).

As the PI3K/AKT and MAPK pathways are major survival and proliferative signalling pathways, respectively, and are commonly constitutively activated in cancer, simultaneous inhibition of these pathways by combined MEK and PI3K inhibition may be required for optimal antitumour activity (Kohno and Pouyssegur, 2006). Additionally, mechanistic data suggest that crosstalk exists between the MAPK and PI3K pathways, and thus to achieve maximal effects inhibition of both pathways may be necessary (Ma *et al*, 2005, 2007). *In-vitro* studies using dual pharmacological inhibition of these pathways have shown that combination treatment augments antiproliferative activity, for example, with combinations of the MEK inhibitor PD0325901 with the PI3K inhibitor LY294002 (Liu and Xing, 2008), or the MEK inhibitors CI-1040 and UO126 with the PI3K inhibitors WAY-266176 and WAY-266175 (Yu *et al*, 2008). However, *in-vivo* combination studies exhibited the most impressive results, for example, synergistic regression was achieved using the PI3K inhibitor NVP-BEZ235 and the MEK inhibitor AZD6244 in mice with KRAS-G12D-induced lung tumours or EGFR mutant tumours (Engelman *et al*, 2008; Faber *et al*, 2009).

In the study described here, combinations of the MEK inhibitors AZD6244 or PD0325901, and the dual mTOR/PI3K inhibitor NVP-BEZ235, or the pan class I PI3K inhibitor GDC-0941, exhibited marked synergistic growth inhibition against human tumour cell lines. However, GDC-0941 produced greater synergy in combination with either MEK inhibitor. Western blotting revealed marked inhibition of 4EBP1 phosphorylation with NVP-BEZ235 alone and in combination, whereas there was minimal inhibition with GDC-0941, suggesting that the dual mTOR/PI3K inhibitory activity of NVP-BEZ235 may underlie its ability to inhibit 4EBP1 phosphorylation. Studies using the mTORC1/2 inhibitor KU0063794 in combination with GDC-0941, confirmed that the decreased synergy exhibited by NVP-BEZ235 in combination with MEK inhibitors, compared with GDC-0941, was due to mTOR inhibition, as low concentrations of KU0063794 were sufficient to compromise the synergy of GDC-0941 with a MEK inhibitor.

## Materials and methods

### Cell lines and reagents

HCT116 (KRas mutant, PIK3CA mutant), DLD1 (KRas mutant, PIK3CA mutant) and HT29 (BRaf mutant, PIK3CA mutant) human colorectal cancer cells were obtained from the American Type Culture Collection, were grown in RPMI-1640 medium (supplemented with 10% (v/v) fetal bovine serum, 1% (v/v) penicillin (50 U ml^−1^)–streptomycin (50 mg ml^−1^) and 2 mM L-glutamine) and confirmed free of mycoplasma contamination by regular testing with Mycoalert (Cambrex, Charles City, IA, USA).

### Inhibitors

The PI3K inhibitors NVP-BEZ235 and GDC-0941, and the MEK inhibitors AZD6244 and PD0325901, were kindly supplied by UCB Celltech, Slough, Berkshire, UK. The mTORC1/2 inhibitor KU0063794 was purchased from Selleck Chemicals, Houston, TX, USA. All drugs were dissolved in dimethyl sulphoxide (DMSO) to allow addition to cell cultures at a final concentration of 0.5% (v/v) DMSO, and were stored frozen under light-protected conditions at −20 °C.

### Growth inhibition assay

Growth inhibition was measured using the Sulforhodamine B (SRB) method as described previously (Skehan *et al*, 1990), and in Supplementary Materials and Methods.

### Cytotoxicity assay

Clonogenic assays were performed as described previously (Kyle *et al*, 2008), and in Supplementary Materials and Methods.

### Western blotting

Western blots were prepared and developed as described in Supplementary Materials and Methods.

## Results

### PI3K and MEK inhibitors are synergistic in combination

The growth inhibitory activity of the PI3K inhibitor GDC-0941, the PI3K/mTOR inhibitor NVP-BEZ235, the mTOR inhibitor KU0063794 and the MEK inhibitors AZD6244 and PD0325901, in HCT116, DLD1 and HT29 cells was measured using the SRB assay (Supplementary Figure S1). All five drugs achieved >75% growth inhibition in the three colorectal cell lines. The results were used to determine the half maximal growth inhibitory concentration (GI_50_) of the five drugs after 72 h exposure. PD0325901 was the most potent MEK inhibitor with GI_50_ values of 21, 1460 and 6.5 nM, compared with 312, 4973 and 29 nM with AZD6244 in the HCT116, DLD1 and HT29 cell lines, respectively. The dual PI3K/mTOR inhibitor NVP-BEZ235 was a markedly more potent inhibitor of growth than either the PI3K inhibitor GDC-0941 or the mTOR inhibitor KU0063794; GI_50_ values of 21, 7.2 and 9.4 nM with NVP-BEZ235 compared with 1081, 1070 and 157 nM with GDC-0941 and 767, 568 and 402 nM with KU0063794 in the HCT116, DLD1 and HT29 cell lines, respectively.

**Figure 1:**
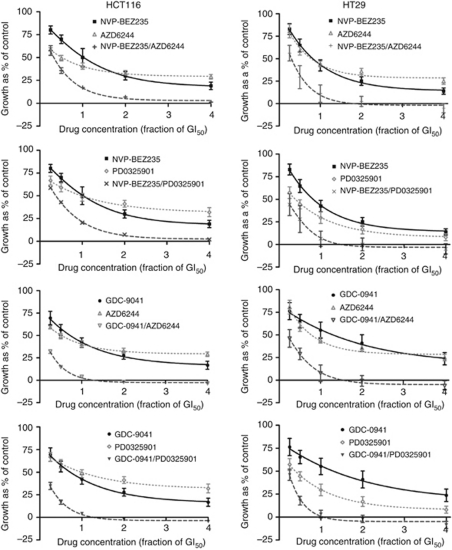
Growth inhibition induced by the PI3K inhibitors, NVP-BEZ235 or GDC-0941, and the MEK inhibitors, AZD6244 or PD0325901, alone and in combination. HCT116 and HT29 cells were treated with the indicated fractions of the GI_50_ concentrations of the inhibitors, alone or in combination, derived from Supplementary Figure S1, for 72 h, and an SRB assay was subsequently performed. Data are presented as a percentage of the control, in which cells were treated with 0.5% (v/v) DMSO. Points represent the mean of three independent experiments±standard error. Lines were fitted using non-linear regression analysis.

Studies were then performed to determine the effect of combined PI3K and MEK inhibitor treatment on HCT116 and HT29 cell growth over 72 h. The PI3K and MEK inhibitors were used alone at 0.25 × , 0.5 × , 1 × , 2 × and 4 × their respective GI_50_ concentration, and at equipotent concentrations at the same ratios in combination. Figure 1 illustrates that, for all four pairs of inhibitors, the combinations were markedly more growth inhibitory than either compound alone, reducing growth to <10% of control at the highest concentrations. Additionally, there appeared to be greater growth inhibition when GDC-0941 was combined with either MEK inhibitor, compared with the combinations with NVP-BEZ235.

**Figure 2:**
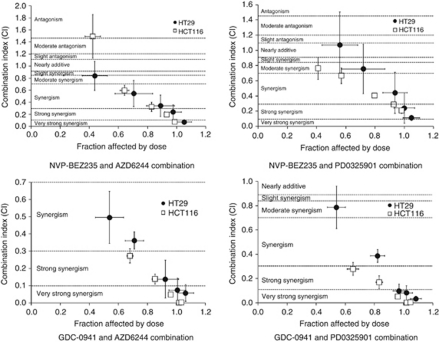
Interaction of PI3K and MEK inhibitor combinations. Median effect analysis (CalcuSyn software) was used to evaluate the interaction between the inhibitor combinations shown in Figure 1. Points represent the mean of three independent experiments±standard error. Horizontal dotted lines indicate the boundaries for each interaction classification.

Data were evaluated by median effect analysis (CalcuSyn, Biosoft, Great Shelford, UK) to determine whether the greater growth inhibitory activity of the combinations reflected an additive or a synergistic effect. All combinations of PI3K and MEK inhibitors were synergistic when combined at the GI_50_ concentration compared with the compounds alone; however, despite NVP-BEZ235 being a more potent PI3K inhibitor, GDC-0941 displayed greater synergy when combined with either MEK inhibitor at a constant ratio of their respective GI_50_ values in the HCT116 and HT29 cell lines (Figure 2; Supplementary Table S1). Similar results were obtained when NVP-BEZ235 and GDC-0941 were combined with PD0325901 in DLD1 cells (Supplementary Figure S2; Supplementary Table S2).

### PI3K and MEK inhibitors alone and in combination are predominantly cytostatic

Cell survival after exposure to the PI3K inhibitors NVP-BEZ235 and GDC-0941, and the MEK inhibitors AZD6244 and PD0325901, was measured using a clonogenic cytotoxicity assay. As a single agent, only NVP-BEZ235 showed significant cytotoxicity at 10 *μ*M, 97% cell kill in the HCT116 cell line and 82% in the HT29 cell line; however, the mean lethal concentration (LC_50_) of 0.5 *μ*M NVP-BEZ235 in both cell lines was ⩾20-fold higher than the corresponding GI_50_ values. The three other compounds induced <50% cell death after 72 h treatment at 10 *μ*M (Supplementary Figure S3).

The cytotoxicity of the PI3K and MEK inhibitors in combination after 72 h treatment was also determined. However, as only NVP-BEZ235 produced >50% cytotoxicity at 10 *μ*M, it was not possible to determine the LC_50_ value of all of the compounds, and hence 10 *μ*M GDC-0941 was combined with 10 *μ*M AZD6244 or 10 *μ*M PD0325901, concentrations above 10 *μ*M not being pharmacologically relevant. In contrast, as NVP-BEZ235 did display cytotoxicity as a single agent, it was combined with 10 *μ*M of the MEK inhibitors at 0.1 *μ*M, the highest non-toxic concentration of single agent NVP-BEZ235, as well as at cytotoxic concentrations of 1 and 10 *μ*M. There was no significant difference in cytotoxicity when the PI3K and MEK inhibitors were combined, compared with the cytotoxicity induced by the drugs as single agents, in the HCT116 cell line or with most combinations in the HT29 cell line. However, the combination of 10 *μ*M GDC-0941 with 10 *μ*M of either MEK inhibitor, and the combination of 0.1 *μ*M NVP-BEZ235 with 10 *μ*M PD0325901 only, did display a statistically significant increase in cytotoxicity in the HT29 cell line (Supplementary Figure S4). Overall, while the synergistic interaction of the PI3K and MEK inhibitors resulted in enhanced cell growth inhibition, there was no consistent increase in cytotoxicity.

### Combinations of PI3K and MEK inhibitors enhance phosphorylation of S6 but have no clear or consistent effects on ERK or 4EBP1 phosphorylation

The effect of 24-h exposure to the PI3K inhibitors NVP-BEZ235 and GDC-0941, and the MEK inhibitors AZD6244 and PD0325901, both as single agents and in combination, was investigated by western blotting to determine the effect on the PI3K/AKT signalling pathway, using total and phospho-specific antibodies for AKT, S6 and 4EBP1. The effect on MAPK signalling was studied using total and phospho-specific antibodies for ERK, and the compounds were used as single agents at their respective GI_50_ concentrations and at 10 × the GI_50_ concentration.

**Figure 3:**
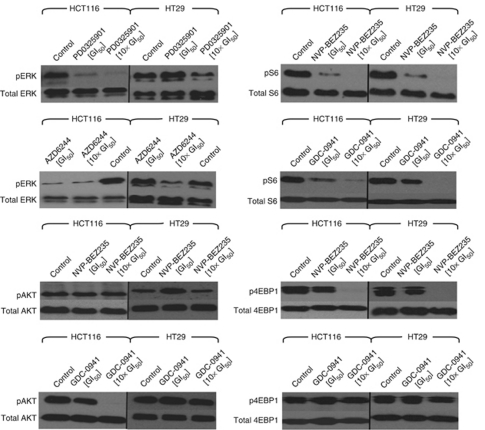
Effect of the MEK inhibitors, PD0325901 and AZD6244, as single agents on MAPK signal transduction and the PI3K inhibitors, NVP-BEZ235 and GDC-0941, as single agents on PI3K/AKT signal transduction. HCT116 and HT29 cells were treated with the indicated concentrations of the inhibitors, derived from Supplementary Figure S1, for 24 h. Cell lysates were subjected to electrophoresis, followed by western blotting using the indicated phospho-specific antibodies. Blots were then stripped and reprobed with the corresponding total antibody to confirm equal protein loading. Data shown are representative of ⩾3 independent experiments.

Figure 3 shows that at 24 h ERK phosphorylation was almost completely inhibited by both PD0325901 and AZD6244 at 1 × and 10 × the GI_50_ concentration in the HCT116 cell line, whereas inhibition of ERK phosphorylation was only observed at 10 × the GI_50_ value in the HT29 cell line with both the MEK inhibitors. The effects of the PI3K inhibitors on AKT phosphorylation at the time point and concentrations studied was limited as NVP-BEZ235 caused no inhibition in either cell line, and there was only inhibition at 10 × the GI_50_ value with GDC-0941 in the HCT116 cell line. In contrast, S6 phosphorylation was markedly inhibited by both NVP-BEZ235 and GDC-0941 in both cell lines. Overall, equipotent growth inhibitory concentrations of the PI3K and MEK inhibitors appear to cause stronger inhibition of the PI3K/mTOR and MAPK signalling pathways, respectively, in the HCT116 cell line than in the HT29 cell line at 24 h. The inhibition of 4EBP1, however, was dependent on the PI3K inhibitor rather than the cell line, as NVP-BEZ235 clearly inhibited 4EBP1 phosphorylation at 10 × the GI_50_ value in both cell lines whereas GDC-0941 showed minimal, if any, inhibition (Figure 3).

The compounds were then used as single agents or in combination at 0.1 × , 1 × and 10 × their respective GI_50_ concentrations using PD0325901 as a representative MEK inhibitor. Table 1 and Supplementary Figure S5 show that there were no major differences between the inhibition of ERK phosphorylation by PD0325901, alone or in combination, in the HCT116 cell line, as pERK was reduced at the GI_50_ value and completed inhibited at 10 × the GI_50_ value, regardless of the presence or absence of the PI3K inhibitor. At 1 × and 10 × GI_50_, there was no inhibition of ERK phosphorylation by PD0325901 alone in the HT29 cell line; however, there was an increase in the inhibition of ERK phosphorylation when PD0325901 was combined with either PI3K inhibitor.

Inhibition of AKT phosphorylation, by the inhibitors alone or in combination, was not pronounced, as there was only inhibition with GDC-0941 in HCT116 cells, but no increase in inhibition with the combination of GDC-0941 and PD0325901 at 10 × the GI_50_ value in either cell line. However, there was an increase in the inhibition of AKT phosphorylation when PD0325901 was combined with NVP-BEZ235 at 10 × the GI_50_ value in HCT116 cells. In contrast to AKT, inhibition of S6 phosphorylation was pronounced, as combinations of PD0325901 with either PI3K inhibitor at 1 × or 10 × the GI_50_ value completely inhibited S6 phosphorylation in both cell lines, showing an increase in inhibition over that observed with the PI3K inhibitor alone where it had not already caused complete inhibition (Table 1; Supplementary Figure S5). Loss of the phospho-4EBP1 signal was observed when NVP-BEZ235 was used, both alone and in combination, at 10 × the GI_50_, in both cell lines. However, GDC-0941 alone had no effect on 4EBP1 phosphorylation, and only a minor effect was observed in combination with PD0325901 in HT29 cells. Overall, the combination of the MEK inhibitor PD0325901 with either PI3K inhibitor resulted in enhanced inhibition of S6 phosphorylation in both cell lines, and increased the inhibition of AKT phosphorylation in combination with NVP-BEZ235 but not with GDC-0941 in the HCT116 cell line. However, importantly, the combinations had no clear or consistent impact on ERK or 4EBP1 phosphorylation over that produced by the agents alone.

### The mTORC1/2 inhibitor KU0063794 compromises the synergy of GDC-0941/PD0325901 combination

The main difference between the two PI3K inhibitors was the ability of NVP-BEZ235, as opposed to GDC-0941, to inhibit 4EBP1 phosphorylation, presumably as a result of direct mTOR inhibition. Hence, it was hypothesised that addition of an mTORC1/2 inhibitor would compromise the synergy observed with the pan PI3K inhibitor GDC-0941. Using median effect analysis, the mTORC1/2 inhibitor KU0063794 was found to produce similar synergy in combination with either MEK inhibitor to that observed with NVP-BEZ235, and interestingly was also found to be synergistic with GDC-0941. However, conversely, KU0063794 was antagonistic in combination with NVP-BEZ235, consistent with both compounds acting on the same target (Supplementary Figure S6; Supplementary Table S3). Additionally, western blotting revealed that the combination of GDC-0941 and KU0063794 inhibited the phosphorylation of 4EBP1 and S6 to a similar extent to that caused by single agent NVP-BEZ235 in HCT116, DLD1 and HT29 cell lines (Supplementary Figure S7).

**Figure 4:**
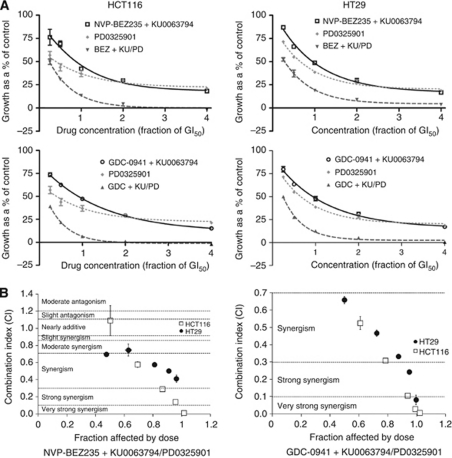
Effect of the PI3K/mTOR inhibitor NVP-BEZ235 or the PI3K inhibitor GDC-0941, mixed at an equipotent concentration with the mTORC1/2 inhibitor KU0063794 in combination with the MEK inhibitor, PD0325901. (**A**) Growth inhibition. HCT116 and HT29 cells were treated with the indicated fractions of the GI_50_ concentration of the mixed PI3K and mTOR inhibitors (data not shown) and the GI_50_ concentration of the MEK inhibitor (calculated from Supplementary Figure S1), alone or in combination for 72 h, and an SRB assay was subsequently performed. Data are presented as a percentage of the control, in which cells were treated with 0.5% (v/v) DMSO. Points represent the mean of three independent experiments±standard error and lines were fitted using non-linear regression analysis. (**B**) Interaction of the combinations. Median effect analysis (CalcuSyn software) was used to evaluate the interaction between the inhibitor combinations. Points represent the mean of three independent experiments±standard error. Horizontal dotted lines indicate the boundaries for each interaction classification.

A GI_50_ value was then determined for the combination of GDC-0941 or NVP-BEZ235 with KU0063794 (data not shown), and these mixed PI3K and mTOR inhibitor combinations were then combined with PD0325901, to determine the effect of dual PI3K/mTOR inhibition on MEK inhibitor synergy. Figure 4 and Supplementary Table S4 indicate that the addition of the mTOR inhibitor compromised the synergy of GDC-0941 and PD0325901 combinations, compared with the GDC-0941/PD0325901 combination alone (Figures 1 and 2; Supplementary Table S1).

**Figure 5:**
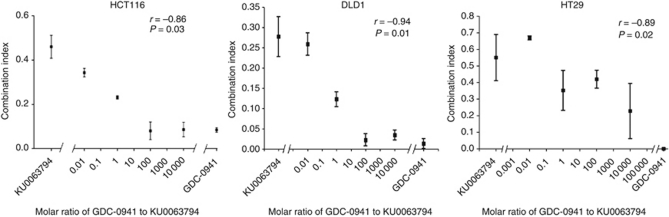
Relationship between the PI3K inhibitor (GDC-0941): mTORC1/2 inhibitor (KU0063794) concentration ratio and synergy with the MEK inhibitor PD0325901 in the HCT116, DLD1 and HT29 cell lines. Cells were treated with the PI3K inhibitor GDC-0941 alone, the mTORC1/2 inhibitor KU0063794 alone or the indicated ratios of the two inhibitors together, in combination with the MEK inhibitor PD0325901 for 72 h, and an SRB assay was subsequently performed. Median effect analysis (CalcuSyn software) was used to evaluate the interaction between the inhibitor combinations. Points represent the mean of the combination index at 1 × the GI_50_ concentration for the HCT116 and HT29 cell lines, and 0.5 × the GI_50_ concentration for the DLD1 cell line, of three independent experiments±standard error. Correlation (*r*) and significance (*P*) were calculated using two-tailed Spearman's Rank Correlation analysis.

To confirm the impact of adding an mTOR inhibitor on the synergistic interaction of GDC-0941 and PD0325901, different molar ratios of GDC-0941 to KU0063794, from 0.01 : 1 to 10 000 : 1, were used in combination with PD0325901 and synergy was evaluated using median effect analysis. The data were then analysed using Spearman's Rank Correlation analysis, which demonstrated a statistically significant positive correlation between increasing ratios of GDC-0941 to KU0063794 in combination with PD0325901, with synergy in the HCT116 and DLD1 cell lines plateauing at ratios ⩾100 : 1 GDC-0941:KU0063794 (Figure 5; Supplementary Tables S5 and S6). Data for the HT29 cell line were more variable; however, there was again a statistically significant impact of KU0063794 on the synergy of GDC-0941 and PD0325901 combinations (Figure 5; Supplementary Table S7). Together, these data demonstrate that even submicromolar concentrations of KU0063794 are sufficient to compromise the synergy of the GDC-0941 and PD0325901 combinations in all three cell lines studied.

## Discussion

Numerous targeted small molecule inhibitors are being developed as a new approach to cancer therapy; however, as tumours often have defects in multiple signalling pathways single agent antitumour activity is modest thus combination treatment is being investigated. The current study has shown that the PI3K inhibitors NVP-BEZ235 and GDC-0941 are synergistic in combination with the MEK inhibitors PD0325901 and AZD6244. However, despite NVP-BEZ235 being a more potent PI3K inhibitor, GDC-0941 showed greater synergy in combination with either MEK inhibitor.

Previous studies using a variety of cell lines report data consistent with the GI_50_ values determined here for the PI3K inhibitors NVP-BEZ235 and GDC-0941, and the MEK inhibitors AZD6244 and PD0325901 (Yeh *et al*, 2007; Folkes *et al*, 2008; Maira *et al*, 2008; Hennig *et al*, 2010), and differences between compounds in relative growth inhibitory potencies are related to the cellular pharmacology, that is, potency and specificity of kinase inhibition, and/or cellular uptake and retention. The PI3K inhibitors are structurally different, and inhibit PI3K family enzymes with different potencies. NVP-BEZ235 is equipotent for mTOR and PI3K (Maira *et al*, 2008), whereas GDC-0941 is 10-fold less active against mTOR in both absolute and relative terms (Folkes *et al*, 2008). Thus, the increased cell growth inhibitory activity of NVP-BEZ235 may be related to its ability to inhibit mTOR (Maira *et al*, 2009). In addition to mTOR, NVP-BEZ235 potently inhibits DNA-PK and class II PI3Ks, which may also increase its ability to inhibit cell growth (Kong *et al*, 2009, 2010). In contrast to the PI3K inhibitors, although AZD6244 and PD0325901 are structurally similar and highly selective for MEK they nevertheless exhibit different potencies for both MEK and cell growth inhibition (Yeh *et al*, 2007; Barrett *et al*, 2008).

All the MEK and PI3K inhibitor combinations tested in this study displayed marked synergistic growth inhibitory activity, which is consistent with other *in-vitro* data in which, for example, PD0325901 exhibited synergy with the PI3K inhibitor LY294002 (Liu and Xing, 2008), and the MEK inhibitors CI-1040 and UO126 showed augmented growth inhibition when combined with the PI3K inhibitors WAY-266175 and WAY-266176 (Yu *et al*, 2008). However, a novel aspect of this study is the observation that GDC-0941 exhibits stronger synergy than NVP-BEZ235 in combination with either MEK inhibitor at equipotent concentrations, which suggests that the mTOR inhibition exhibited by NVP-BEZ235 may limit synergistic growth inhibitory activity when combined with MEK inhibitors. As both the PI3K and MAPK pathways inhibit TSC2, which is directly upstream of mTORC1 (Ma *et al*, 2005), the increased synergy exhibited by PI3K and MEK inhibition may be due to increased inhibition of mTORC1. Thus, the ability of single agent NVP-BEZ235 to potently inhibit mTOR may account for the decreased synergy observed in combination with a MEK inhibitor.

Whereas all of the compounds were growth inhibitory, only NVP-BEZ235 was cytotoxic to HCT116 and HT29 cells, with an LC_50_ of 0.5 *μ*M, which could be due to the inhibition of mTOR either alone or in combination with PI3K. These results are consistent with other studies, for example, NVP-BEZ235 was found to be cytotoxic to breast cancer cell lines (Serra *et al*, 2008), whereas MEK inhibitors were reported to be cytostatic (Kohno and Pouyssegur, 2006). However, another study reported that AZD6244 caused apoptosis in some human tumour xenografts but did not in others, which is suggested to be due to differences in ERK substrate expression or differential cell signalling networks in tumour cells (Davies *et al*, 2007). Hence, overall, these data suggest that the cytotoxicity exhibited by MEK and PI3K inhibitors depends on the cell line used; consistent with the view of Maira *et al*, who noted that cell death induction is not always observed with NVP-BEZ235 (Maira *et al*, 2009), and that PTEN-null cell lines are potentially more susceptible to the cytotoxic effects of the compound (Maira *et al*, 2008).

The marked synergy observed in the growth inhibition studies did not translate into cytotoxicity, as the effect of the combinations was no greater than that observed with the compounds alone in the HCT116 cell line, although some combinations involving GDC-0941 in combination with either MEK inhibitor and the lowest concentration of NVP-BEZ235 in combination with PD0325901 did show a small and statistically significant difference in cytotoxicity in HT29 cells. Where enhanced cytotoxicity was observed this could be due to increased inhibition of mTORC1 as combined inhibition of both pathways relieves the AKT- and ERK-mediated inhibition of TSC2 (Ma *et al*, 2005). However, this mTORC1 inhibition by the combinations may be incomplete in comparison with NVP-BEZ235, which would explain why there was no statistically significant increase in cytotoxicity in the HCT116 cell line. In contrast to the results in the HCT116 cell line, one *in-vitro* study concluded that combined inhibition of PI3K and ERK, induced by PIK3CA downregulation and PD0325901, respectively, was at least in part due to the induction of cell death (Wee *et al*, 2009), and an *in-vivo* study in mice with tumours carrying PI3K and Ras mutations demonstrated synergistic tumour regression with NVP-BEZ235 and AZD6244 (Engelman *et al*, 2008). However, the effect of gene downregulation is potentially different to the effects of small molecule inhibition, and in *in-vivo* studies processes such as antiangiogenic effects may also be important determinants of response, as suggested by Maira *et al* (2009).

In the cell signalling investigations, PD0325901 completely ablated ERK phosphorylation at 21 and 6.5 nM, whereas 312 and 29 nM AZD6244 were required to produce substantial inhibition of ERK phosphorylation in the HCT116 and HT29 cell lines, respectively, consistent with the relative growth inhibitory potencies of the compounds. These results are similar to previous reports that ERK phosphorylation was completely inhibited by 200 nM AZD6244 in all cell lines tested (Yeh *et al*, 2007). Interestingly, the range of the growth inhibitory effects of PD0325901 and AZD6244 (15-fold and 5-fold in the HCT116 and HT29 cell lines, respectively) is consistent with the relative potencies of the compounds as MEK inhibitors, implying that any differences in cellular uptake or retention do not affect relative growth inhibitory activity. Importantly, the use of PD0325901 in combination with either PI3K inhibitor demonstrated that there were no marked effects on ERK phosphorylation that could not be ascribed to the effects of PD0325901 alone.

The dual PI3K/mTOR inhibitor NVP-BEZ235 potently inhibited S6 phosphorylation, a well-defined downstream target of both PI3K and mTOR in the PI3K/AKT signalling pathway, consistent with a study by Serra *et al* (2008); in which S6 phosphorylation was suppressed by NVP-BEZ235 in the BT474 and SKBR3 breast cancer cell lines with varying potency. Importantly, the data presented here show definite potentiation by PD0325901 of the inhibition of S6 phosphorylation produced by both PI3K inhibitors.

In contrast to effects on S6 phosphorylation, inhibition of AKT phosphorylation was somewhat less pronounced with no effect after a 24-h exposure to 1 × or 10 × the GI_50_ concentration of either PI3K inhibitor in the HT29 cell line, and only at 10 × GI_50_ concentration of GDC-0941 in the HCT116 cell line. This result is consistent with some previous studies where AKT phosphorylation had recovered by 24 h (Serra *et al*, 2008; Zitzmann *et al*, 2010), whereas others have reported inhibition of AKT phosphorylation after 24 h but only when cells were serum starved and/or stimulated with IGF (Edgar *et al*, 2010; Santiskulvong *et al*, 2011).

In comparison with S6 phosphorylation, higher concentrations of NVP-BEZ235 were required to inhibit 4EBP1 phosphorylation. Nevertheless, at 210 nM in the HCT116 cell line and 94 nM in the HT29 cell line, NVP-BEZ235 completely inhibited 4EBP1 phosphorylation consistent with the inhibition induced by 100 and 500 nM NVP-BEZ235 in the H929 and OPM-2 myeloma cell lines (Baumann *et al*, 2009). However, there was no additional inhibition of 4EBP1 phosphorylation when NVP-BEZ235 was combined with PD0325901. In contrast to NVP-BEZ235, GDC-0941 did not inhibit 4EBP1 phosphorylation at all, either alone or in combination with a MEK inhibitor, which suggests that the ability of NVP-BEZ235 to inhibit mTOR may be responsible for the inhibition of 4EBP1 phosphorylation. Consistent with this theory, the combination of GDC-0941 with KU0063794 inhibited the phosphorylation of both 4EBP1 and S6 to a similar extent to that of NVP-BEZ235 alone. Mechanistically, this theory is reinforced by examination of the PI3K/AKT pathway, as S6 and 4EBP1 are proximal substrates of mTORC1 that is downstream of PI3K.

To test the hypothesis that mTOR inhibition can impair the synergy of PI3K and MEK inhibitors, increasing concentrations of the mTORC1/2 inhibitor KU0063794 were added to the GDC-0941/PD0325901 combination. As predicted, the addition of KU0063794 was found to decrease the synergy of the GDC-0941 and PD0325901 combination; and these data suggest that by inhibiting 4EBP1 phosphorylation and thus preventing the synthesis of proteins that negatively regulate cell-cycle progression, the synergy seen with PI3K/MEK inhibitor combinations is compromised. In contrast, a more selective PI3K inhibitor, such as GDC-0941, is more strongly synergistic in combination with a MEK inhibitor. Additionally, the synergy in the combination of GDC-0941 with a MEK inhibitor may be due to the convergence of the MAPK and PI3K pathways at the level of TSC2, which leads to increased mTORC1 inhibition, whereas the dual PI3K/mTOR inhibitor NVP-BEZ235 is already potently inhibiting mTORC1; and thus, the synergy with a MEK inhibitor is not realised to the same extent.

In conclusion, these studies confirm that dual targeting of PI3K and MEK can induce synergistic growth inhibition. However, despite the dual mTOR/PI3K inhibitor NVP-BEZ235 being more potent as a single agent than the specific PI3K inhibitor GDC-0941, the combination of GDC-0941 with either MEK inhibitor was more synergistic than the combination with NVP-BEZ235.

## Figures and Tables

**Table 1:** Summary of the effect of the PI3K inhibitors, NVP-BEZ235 or GDC-0941, in combination with the MEK inhibitor PD0325901 on protein phosphorylation

	**NVP-BEZ235/PD0325901**		**GDC-0941/PD0325901**	
	**HCT116**	**HT29**	**HCT116**	**HT29**
Combination index at 1 × GI_50_	0.376	0.441	0.045	0.097
				
*Phosphorylation inhibition at 1 × GI*_*50*_				
pERK	++ → ++	− → −	++ → ++	− → ++
pAKT	− → −	− → −	+ → −	− → −
pS6	+ → +++	++ → +++	++ → +++	+ → +++
p4EBP1	+ → +	+ → +	− → −	− → −
				
*Phosphorylation inhibition at 10 × GI*_*50*_				
pERK	+++ → +++	− → ++	+++ → +++	− → +++
pAKT	− → ++	− → −	+ → +	− → −
pS6	++ → +++	+++ → +++	++ → +++	+++ → +++
p4EBP1	+++ → +++	+++ → +++	− → −	+ → +

Abbreviation: GI_50_=half maximal growth inhibitory concentration.

The effects of GDC-0941 or NVP-BEZ235, alone and in combination with PD0325901, on MAPK and PI3K/AKT signal transduction in the HCT116 and HT29 cell lines were summarised from data in Supplementary Figure S5. Data are presented as the effect of the most potent single agent, that is, the PI3K or MEK inhibitor, alone with the arrow pointing to the effect of the combination. Effects on protein phosphorylation are summarised as: −, no effect; +, minimal inhibition; ++, moderate inhibition; +++, complete inhibition.

## References

[bib1] Barrett SD, Bridges AJ, Dudley DT, Saltiel AR, Fergus JH, Flamme CM, Delaney AM, Kaufman M, LePage S, Leopold WR, Przybranowski SA, Sebolt-Leopold J, Van Becelaere K, Doherty AM, Kennedy RM, Marston D, Howard Jr WA, Smith Y, Warmus JS, Tecle H (2008) The discovery of the benzhydroxamate MEK inhibitors CI-1040 and PD 0325901. Bioorg Med Chem Lett 18(24): 6501–6504 18952427 10.1016/j.bmcl.2008.10.054

[bib2] Baumann P, Mandl-Weber S, Oduncu F, Schmidmaier R (2009) The novel orally bioavailable inhibitor of phosphoinositol-3-kinase and mammalian target of rapamycin, NVP-BEZ235, inhibits growth and proliferation in multiple myeloma. Exp Cell Res 315(3): 485–497 19071109 10.1016/j.yexcr.2008.11.007

[bib3] Bodoky G, Timcheva C, Spigel D, La Stella P, Ciuleanu T, Pover G, Tebbutt NC (2011) A phase II open-label randomized study to assess the efficacy and safety of selumetinib (AZD6244 [ARRY-142886]) versus capecitabine in patients with advanced or metastatic pancreatic cancer who have failed first-line gemcitabine therapy. Invest New Drugs 1–8 21594619 10.1007/s10637-011-9687-4

[bib4] Bos JL (1989) Ras oncogenes in human cancer: a review. Cancer Res 49(17): 4682–4689 2547513

[bib5] Davies BR, Logie A, McKay JS, Martin P, Steele S, Jenkins R, Cockerill M, Cartlidge S, Smith PD (2007) AZD6244 (ARRY-142886), a potent inhibitor of mitogen-activated protein kinase/extracellular signal-regulated kinase kinase 1/2 kinases: mechanism of action *in vivo*, pharmacokinetic/pharmacodynamic relationship, and potential for combination in preclinical models. Mol Cancer Ther 6(8): 2209–2219 17699718 10.1158/1535-7163.MCT-07-0231

[bib6] Davies H, Bignell GR, Cox C, Stephens P, Edkins S, Clegg S, Teague J, Woffendin H, Garnett MJ, Bottomley W, Davis N, Dicks E, Ewing R, Floyd Y, Gray K, Hall S, Hawes R, Hughes J, Kosmidou V, Menzies A, Mould C, Parker A, Stevens C, Watt S, Hooper S, Wilson R, Jayatilake H, Gusterson BA, Cooper C, Shipley J, Hargra D, Pritchard-Jones K, Maitland N, Chenevix-Trench G, Riggins GJ, Bigner DD, Palmieri G, Cossu A, Flanagan A, Nicholson A, Ho JW, Leung SY, Yuen ST, Weber BL, Seigler HF, Darrow TL, Paterson H, Marais R, Marshall CJ, Wooster R, Stratton MR, Futreal PA (2002) Mutations of the BRAF gene in human cancer. Nature 417(6892): 949–954 12068308 10.1038/nature00766

[bib7] Edgar KA, Wallin JJ, Berry M, Lee LB, Prior WW, Sampath D, Friedman LS, Belvin M (2010) Isoform-specific phosphoinositide 3-kinase inhibitors exert distinct effects in solid tumors. Cancer Res 70(3): 1164–1172 20103642 10.1158/0008-5472.CAN-09-2525

[bib8] Engelman JA, Chen L, Tan X, Crosby K, Guimaraes AR, Upadhyay R, Maira M, McNamara K, Perera SA, Song Y, Chirieac LR, Kaur R, Lightbown A, Simendinger J, Li T, Padera RF, García-Echeverría C, Weissleder R, Mahmood U, Cantley LC, Wong KK (2008) Effective use of PI3K and MEK inhibitors to treat mutant Kras G12D and PIK3CA H1047R murine lung cancers. Nat Med 14(12): 1351–1356 19029981 10.1038/nm.1890 PMC2683415

[bib9] Faber AC, Li D, Song Y, Liang M-C, Yeap BY, Bronson RT, Lifshits E, Chen Z, Maira SM, García-Echeverría C, Wong KK, Engelman JA (2009) Differential induction of apoptosis in HER2 and EGFR addicted cancers following PI3K inhibition. Proc Natl Acad Sci USA 106(46): 19503–19508 19850869 10.1073/pnas.0905056106 PMC2765921

[bib10] Folkes AJ, Ahmadi K, Alderton WK, Alix S, Baker SJ, Box G, Chuckowree IS, Clarke PA, Depledge P, Eccles SA, Friedman LS, Hayes A, Hancox TC, Kugendradas A, Lensun L, Moore P, Olivero AG, Pang J, Patel S, Pergl-Wilson GH, Raynaud FI, Robson A, Saghir N, Salphati L, Sohal S, Ultsch MH, Valenti M, Wallweber HJ, Wan NC, Wiesmann C, Workman P, Zhyvoloup A, Zvelebil MJ, Shuttleworth SJ (2008) The identification of 2-(1H-Indazol-4-yl)-6-(4-methanesulfonyl-piperazin-1-ylmethyl)-4-morpholin-4-yl-thieno[3,2-d]pyrimidine (GDC-0941) as a potent, selective, orally bioavailable inhibitor of class I PI3 kinase for the treatment of cancer. J Med Chem 51(18): 5522–5532 18754654 10.1021/jm800295d

[bib11] Haura EB, Ricart AD, Larson TG, Stella PJ, Bazhenova L, Miller VA, Cohen RB, Eisenberg PD, Selaru P, Wilner KD, Gadgeel SM (2010) A phase II study of PD-0325901, an oral MEK inhibitor, in previously treated patients with advanced non-small cell lung cancer. Clin Cancer Res 16(8): 2450–2457 20332327 10.1158/1078-0432.CCR-09-1920

[bib12] Hennig M, Yip-Schneider MT, Wentz S, Wu H, Hekmatyar SK, Klein P, Bansal N, Schmidt CM (2010) Targeting mitogen-activated protein kinase kinase with the inhibitor PD0325901 decreases hepatocellular carcinoma growth *in vitro* and in mouse model systems. Hepatology 51(4): 1218–1225 20112426 10.1002/hep.23470

[bib13] Kohno M, Pouyssegur J (2006) Targeting the ERK signaling pathway in cancer therapy. Ann Med 38(3): 200–211 16720434 10.1080/07853890600551037

[bib14] Kong D, Dan S, Yamazaki K, Yamori T (2010) Inhibition profiles of phosphatidylinositol 3-kinase inhibitors against PI3K superfamily and human cancer cell line panel JFCR39. Eur J Cancer 46(6): 1111–1121 20129775 10.1016/j.ejca.2010.01.005

[bib15] Kong D, Yaguchi S-i, Yamori T (2009) Effect of ZSTK474, a novel phosphatidylinositol 3-kinase inhibitor, on DNA-dependent protein kinase. Biol Pharm Bull 32(2): 297–300 19182393 10.1248/bpb.32.297

[bib16] Kyle S, Thomas HD, Mitchell J, Curtin NJ (2008) Exploiting the Achilles heel of cancer: the therapeutic potential of poly(ADP-ribose) polymerase inhibitors in BRCA2-defective cancer. Br J Radiol 81(Spec No 1): S6–11 18820000 10.1259/bjr/99111297

[bib17] Liu D, Xing M (2008) Potent inhibition of thyroid cancer cells by the MEK inhibitor PD0325901 and its potentiation by suppression of the PI3K and NF-κB pathways. Thyroid 18(8): 853–864 18651802 10.1089/thy.2007.0357 PMC2857450

[bib18] Ma L, Chen Z, Erdjument-Bromage H, Tempst P, Pandolfi PP (2005) Phosphorylation and functional inactivation of TSC2 by Erk: implications for tuberous sclerosis and cancer pathogenesis. Cell 121(2): 179–193 15851026 10.1016/j.cell.2005.02.031

[bib19] Ma L, Teruya-Feldstein J, Bonner P, Bernardi R, Franz DN, Witte D, Cordon-Cardo C, Pandolfi PP (2007) Identification of S664 TSC2 phosphorylation as a marker for extracellular signal-regulated kinase–mediated mTOR activation in tuberous sclerosis and human cancer. Cancer Res 67(15): 7106–7112 17671177 10.1158/0008-5472.CAN-06-4798

[bib20] Maira S-M, Stauffer F, Schnell C, Garcia-echeverria C (2009) PI3K inhibitors for cancer treatment: where do we stand? Biochem Soc Trans 37(1): 265–272 19143644 10.1042/BST0370265

[bib21] Maira S-M, Stauffer Fdr, Brueggen J, Furet P, Schnell C, Fritsch C, Brachmann S, Chène P, De Pover A, Schoemaker K, Fabbro D, Gabriel D, Simonen M, Murphy L, Finan P, Sellers W, García-Echeverría C (2008) Identification and characterization of NVP-BEZ235, a new orally available dual phosphatidylinositol 3-kinase/mammalian target of rapamycin inhibitor with potent *in vivo* antitumor activity. Mol Cancer Ther 7(7): 1851–1863 18606717 10.1158/1535-7163.MCT-08-0017

[bib22] Raynaud FI, Eccles SA, Patel S, Alix S, Box G, Chuckowree I, Folkes A, Gowan S, De Haven Brandon A, Di Stefano F, Hayes A, Henley AT, Lensun L, Pergl-Wilson G, Robson A, Saghir N, Zhyvoloup A, McDonald E, Sheldrake P, Shuttleworth S, Valenti M, Wan NC, Clarke PA, Workman P (2009) Biological properties of potent inhibitors of class I phosphatidylinositide 3-kinases: from PI-103 through PI-540, PI-620 to the oral agent GDC-0941. Mol Cancer Ther 8(7): 1725–1738 19584227 10.1158/1535-7163.MCT-08-1200 PMC2718129

[bib23] Santiskulvong C, Konecny GE, Fekete M, Chen K-YM, Karam A, Mulholland D, Eng C, Wu H, Song M, Dorigo O (2011) Dual targeting of phosphoinositide 3-kinase and mammalian target of rapamycin using NVP-BEZ235 as a novel therapeutic approach in human ovarian carcinoma. Clin Cancer Res 17(8): 2373–2384 21372221 10.1158/1078-0432.CCR-10-2289 PMC3078990

[bib24] Sarbassov DD, Guertin DA, Ali SM, Sabatini DM (2005) Phosphorylation and regulation of Akt/PKB by the Rictor-mTOR complex. Science 307(5712): 1098–1101 15718470 10.1126/science.1106148

[bib25] Serra V, Markman B, Scaltriti M, Eichhorn PJA, Valero V, Guzman M, Botero ML, Llonch E, Atzori F, Di Cosimo S, Maira M, Garcia-Echeverria C, Parra JL, Arribas J, Baselga J (2008) NVP-BEZ235, a dual PI3K/mTOR inhibitor, prevents PI3K signaling and inhibits the growth of cancer cells with activating PI3K mutations. Cancer Res 68(19): 8022–8030 18829560 10.1158/0008-5472.CAN-08-1385

[bib26] Skehan P, Storeng R, Scudiero D, Monks A, McMahon J, Vistica D, Warren JT, Bokesch H, Kenney S, Boyd MR (1990) New colorimetric cytotoxicity assay for anticancer-drug screening. J Natl Cancer Inst 82(13): 1107–1112 2359136 10.1093/jnci/82.13.1107

[bib27] Wee S, Jagani Z, Xiang KX, Loo A, Dorsch M, Yao Y-M, Sellers WR, Lengauer C, Stegmeier F (2009) PI3K pathway activation mediates resistance to MEK inhibitors in KRAS mutant cancers. Cancer Res 69(10): 4286–4293 19401449 10.1158/0008-5472.CAN-08-4765

[bib28] Yeh TC, Marsh V, Bernat BA, Ballard J, Colwell H, Evans RJ, Parry J, Smith D, Brandhuber BJ, Gross S, Marlow A, Hurley B, Lyssikatos J, Lee PA, Winkler JD, Koch K, Wallace E (2007) Biological characterization of ARRY-142886 (AZD6244), a potent, highly selective mitogen-activated protein kinase kinase 1/2 inhibitor. Clin Cancer Res 13(5): 1576–1583 17332304 10.1158/1078-0432.CCR-06-1150

[bib29] Yu K, Toral-Barza L, Shi C, Zhang WG, Zask A (2008) Response and determinants of cancer cell susceptibility to PI3K inhibitors: combined targeting of PI3K and Mek1 as an effective cancer strategy. Cancer Biol Ther 7(2): 310–318 10.4161/cbt.7.2.5334 18059185

[bib30] Yuan TL, Cantley LC (2008) PI3K pathway alterations in cancer: variations on a theme. Oncogene 27(41): 5497–5510 18794884 10.1038/onc.2008.245 PMC3398461

[bib31] Zitzmann K, Rüden J, Brand S, Göke B, Lichtl J, Spöttl G, Auernhammer CJ (2010) Compensatory activation of Akt in response to mTOR and Raf inhibitors - a rationale for dual-targeted therapy approaches in neuroendocrine tumor disease. Cancer Lett 295(1): 100–109 20356670 10.1016/j.canlet.2010.02.018

