# Supplementary material for: The synergistic interaction of MEK and PI3K inhibitors is modulated by mTOR inhibition
Source: Br J Cancer. 2012 Mar 13;106(8):1386–94. doi: 10.1038/bjc.2012.70 (PMC3326670; doi:10.1038/bjc.2012.70)
Supplement: Supplementary Information [file bjc201270x1.doc]

**Supplementary Figure S1**

**HCT116**

**
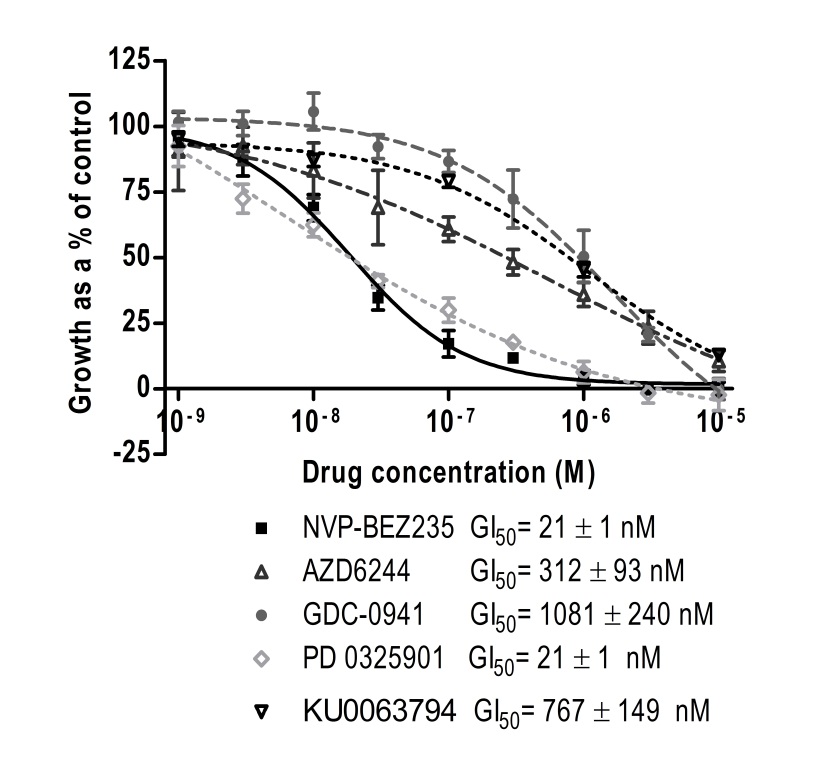
**

**DLD1
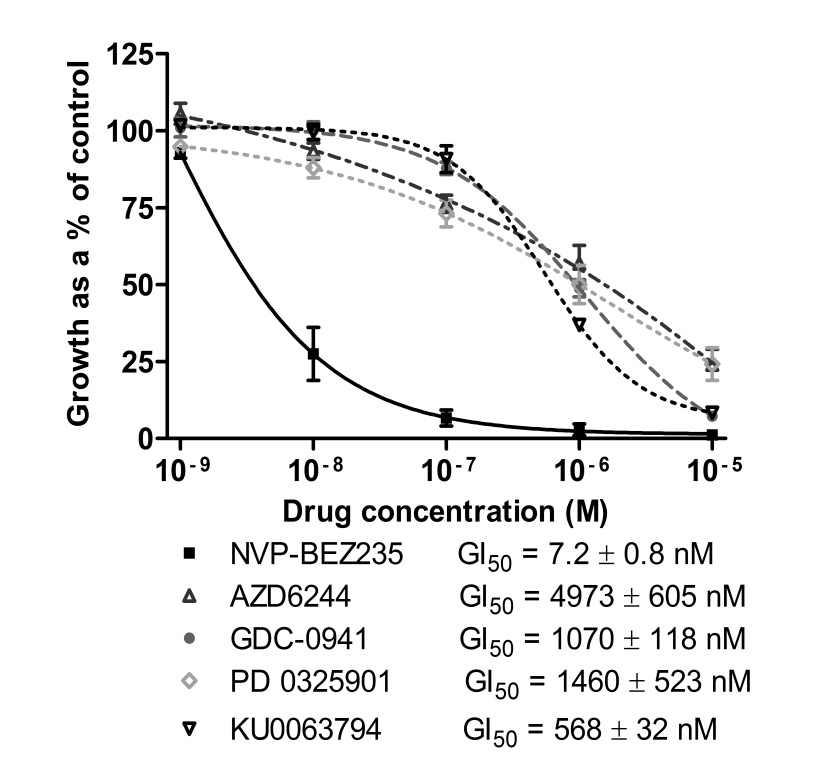
**

**HT29**

**
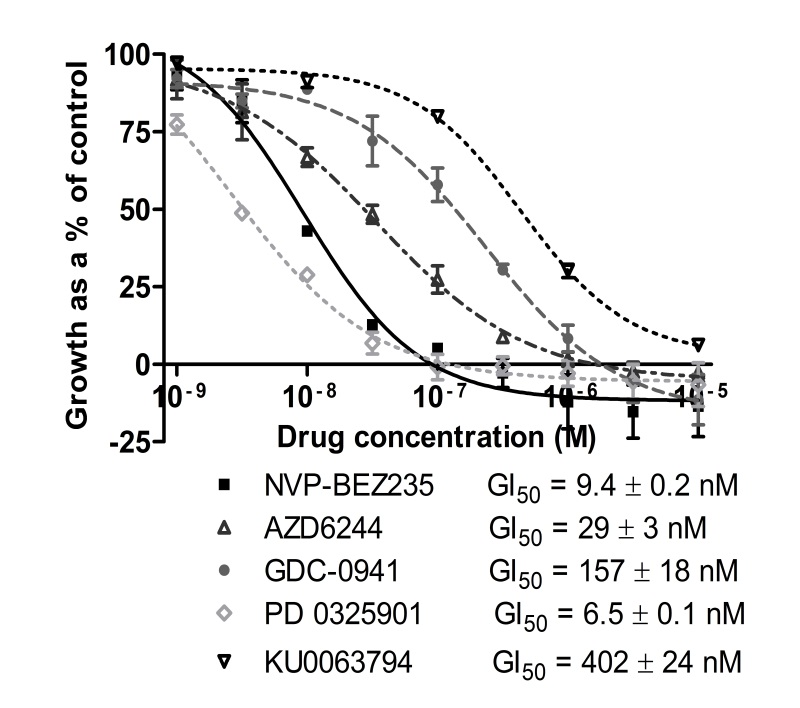
**

**Supplementary Figure S3**

**HCT116**


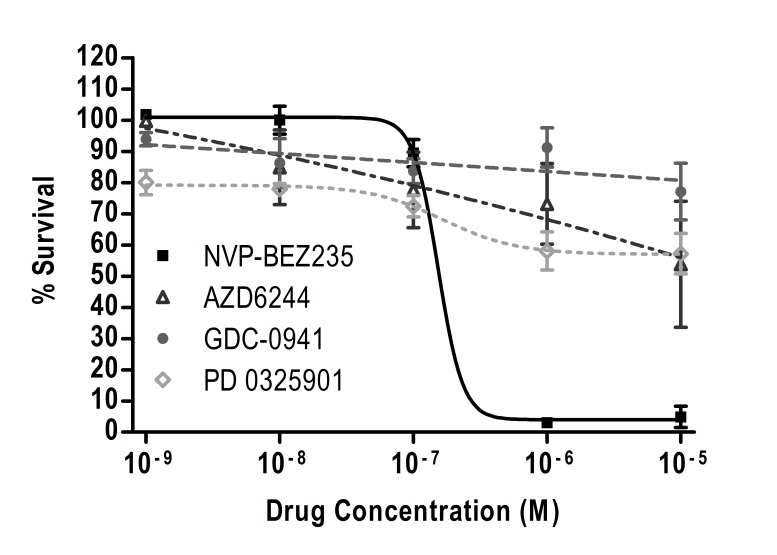


**HT29
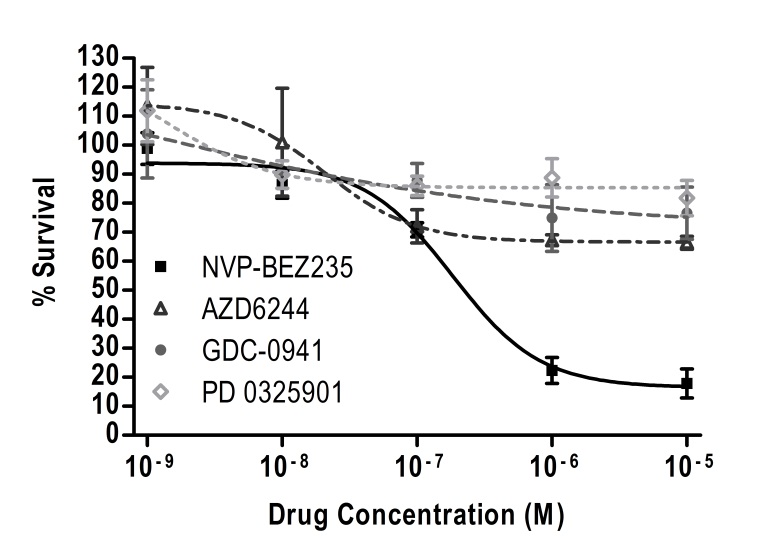
**

**Supplementary Figure S4**

**HCT116**

**
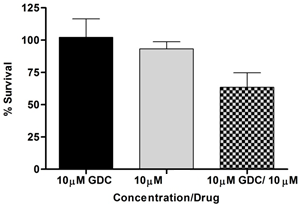

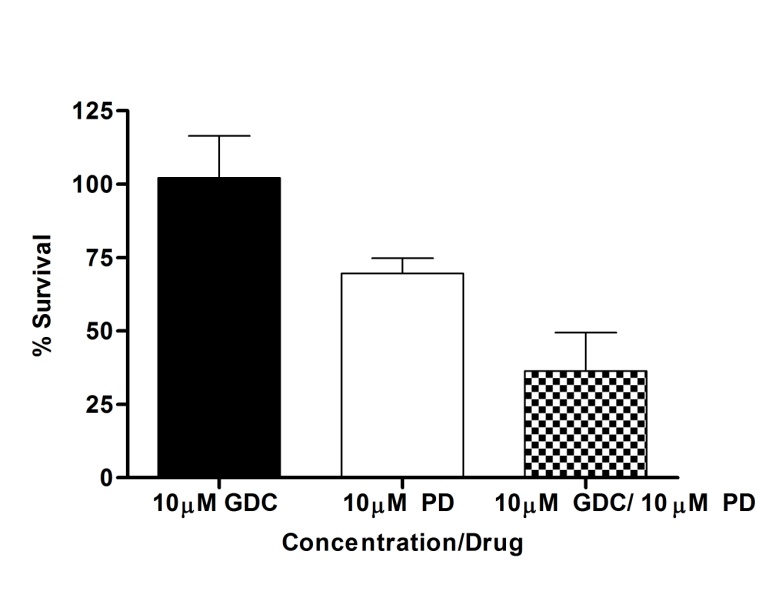

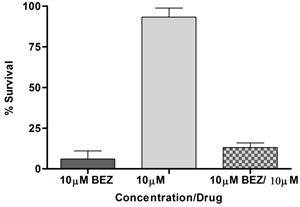

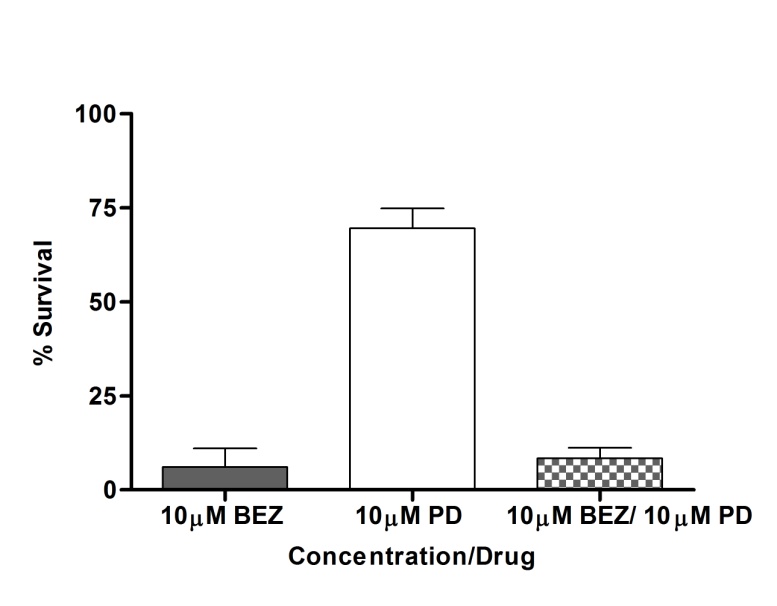
**

**AZD**

**AZD**

**PD**

**PD**

**AZD**

**AZD**

**HT29**

**
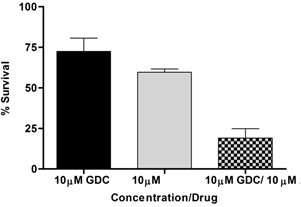

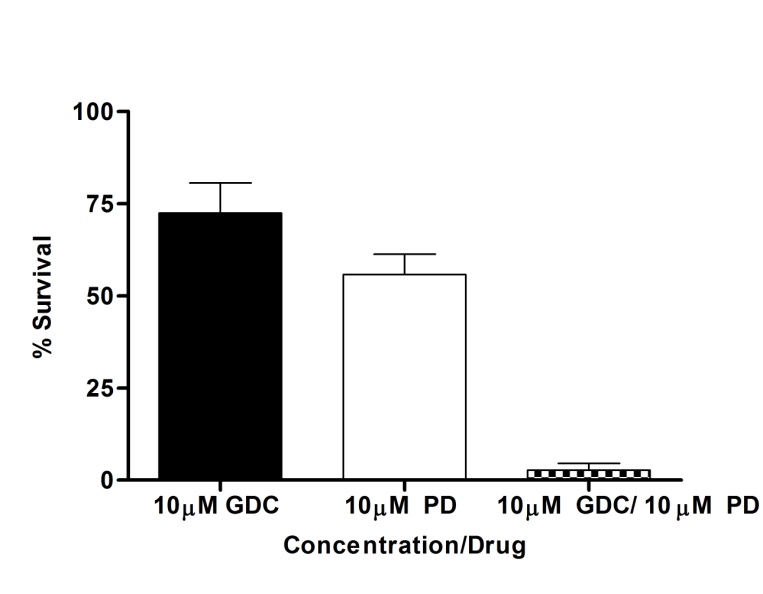

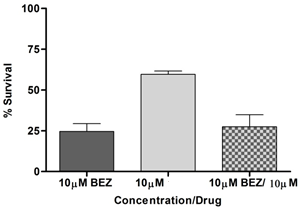

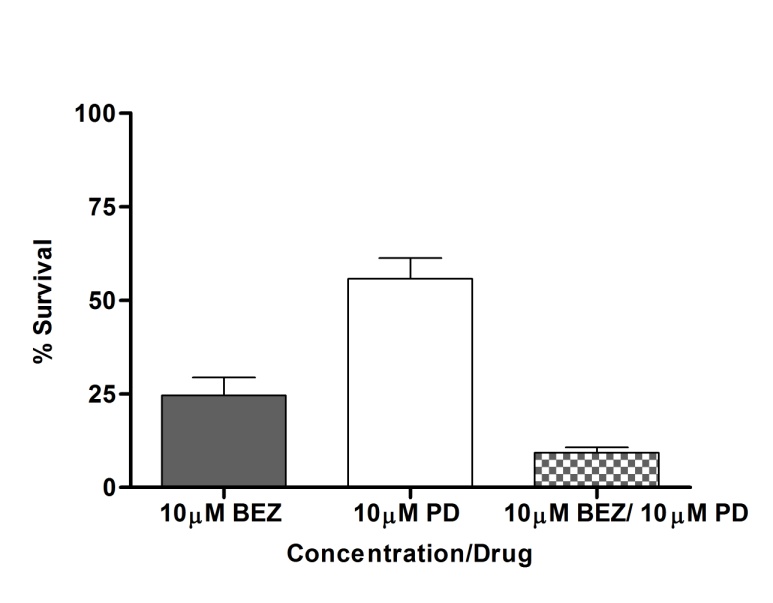
**

**AZD**

**AZD**

**PD**

**PD**

*

*

**AZD**

**AZD**

**HCT116**

**
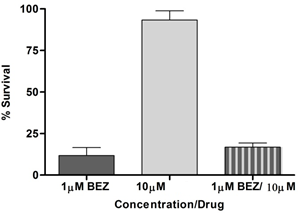

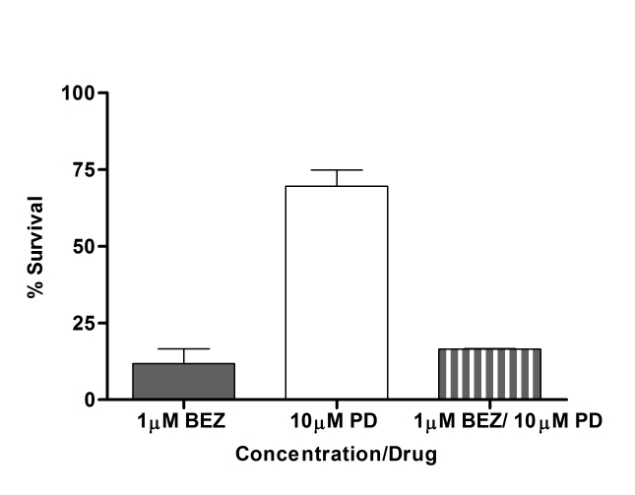

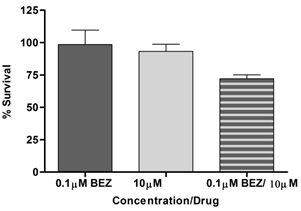

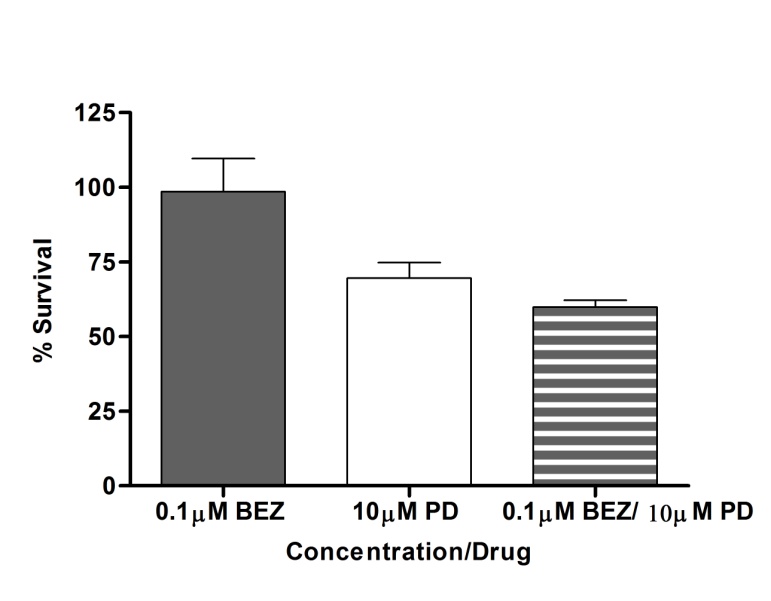
**

**PD**

**PD**

**AZD**

**AZD**

**AZD**

**AZD**

**HT29**

**
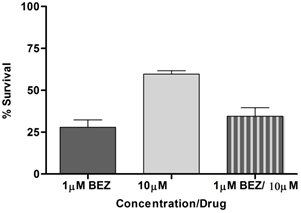

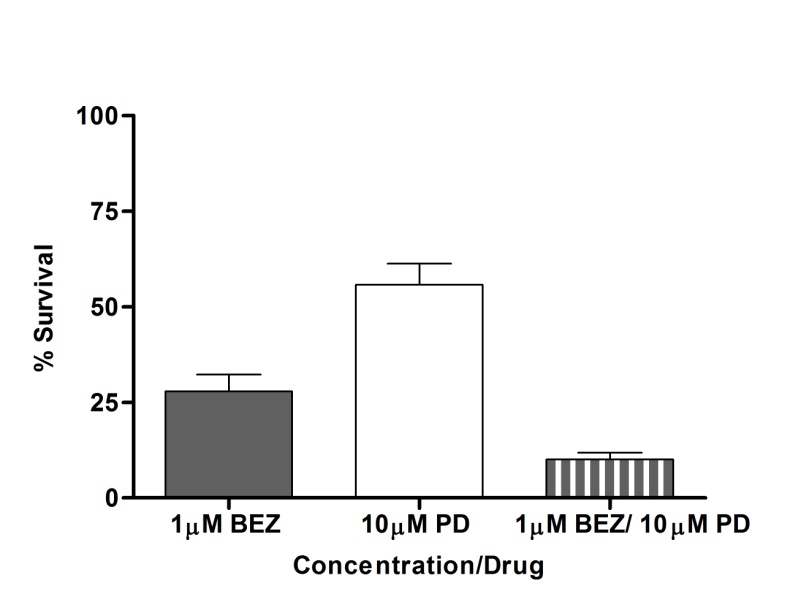
**

**PD**

**AZD**

**AZD**

**
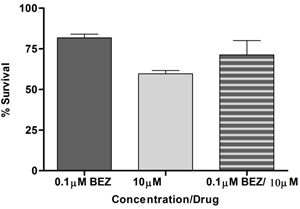

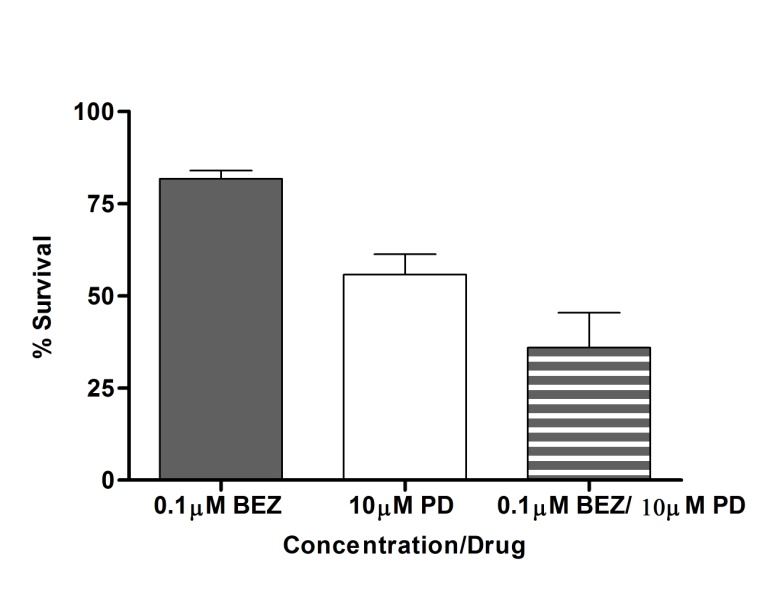
**

**PD**

**AZD**

*

**AZD**

**Supplementary Figure S6**

**
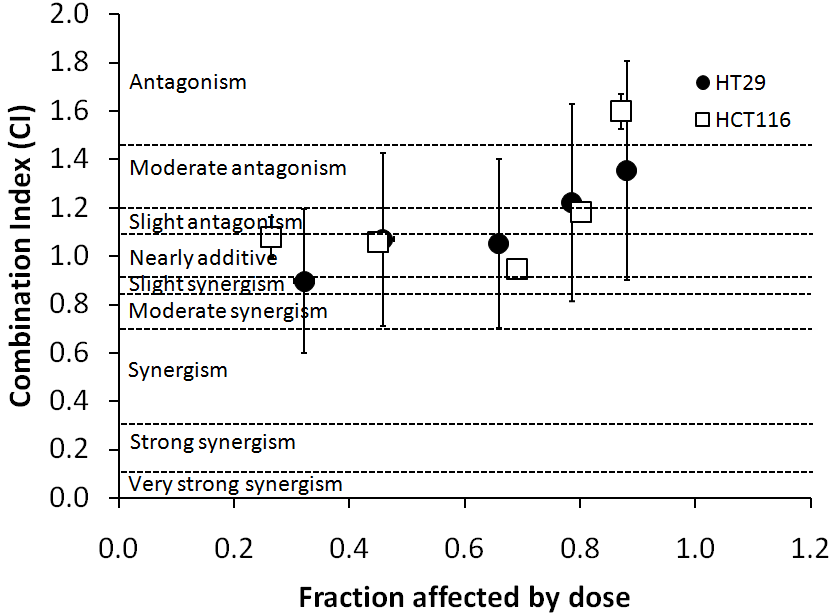
NVP-BEZ235 and KU0063794 Combination**

**
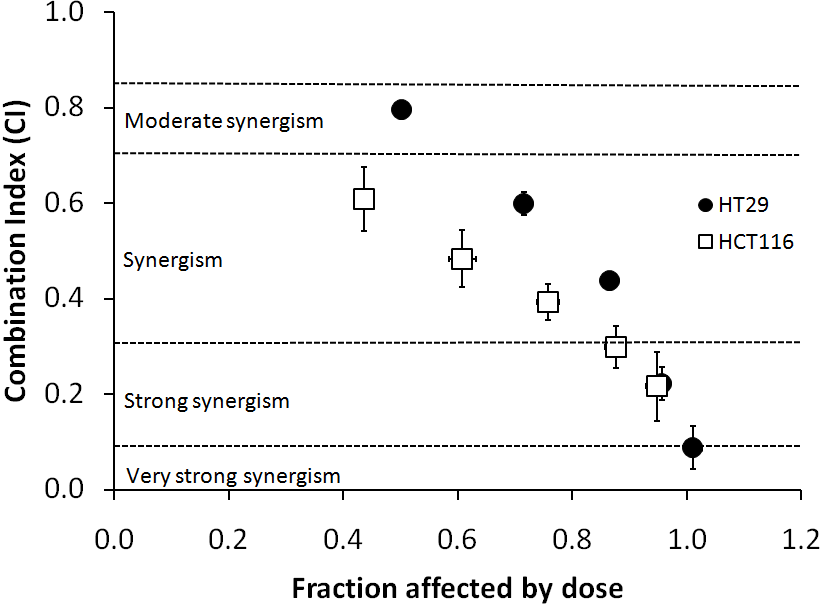
PD 0325901 and KU0063794 Combination**

**
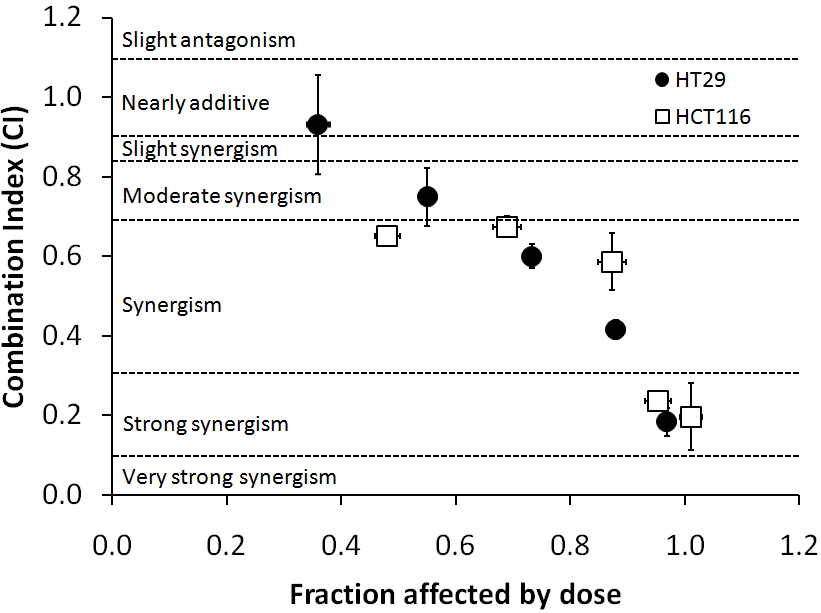
GDC-0941 and KU0063794 Combination**


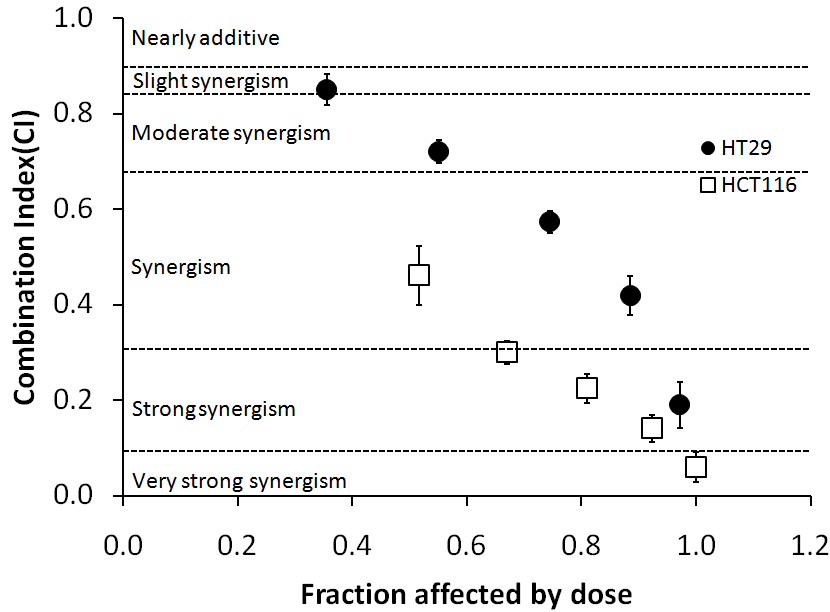
**AZD6244 and KU0063794 Combination**

**Supplementary Figure S5**

**HCT116**

**
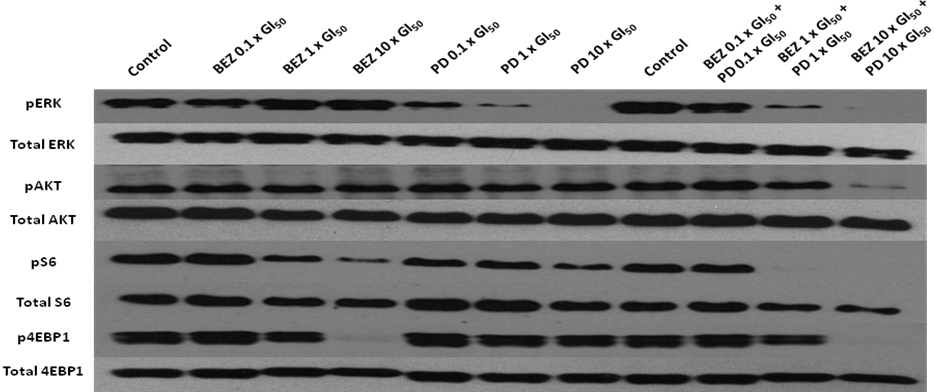
**

**HT29**


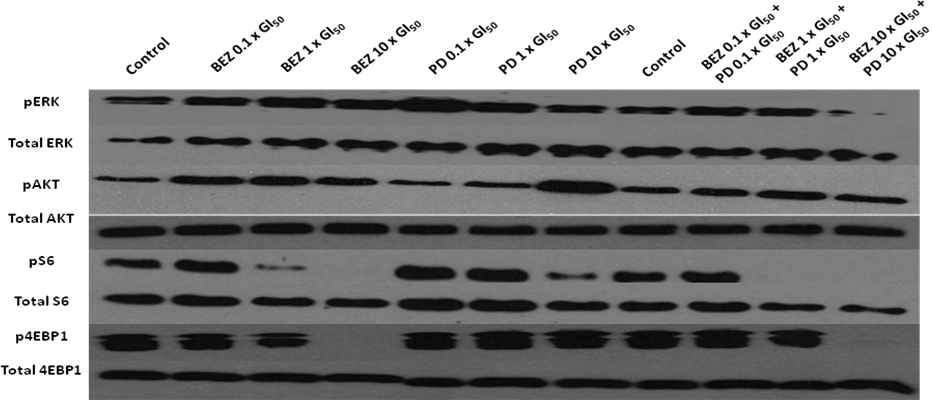


**HCT116**


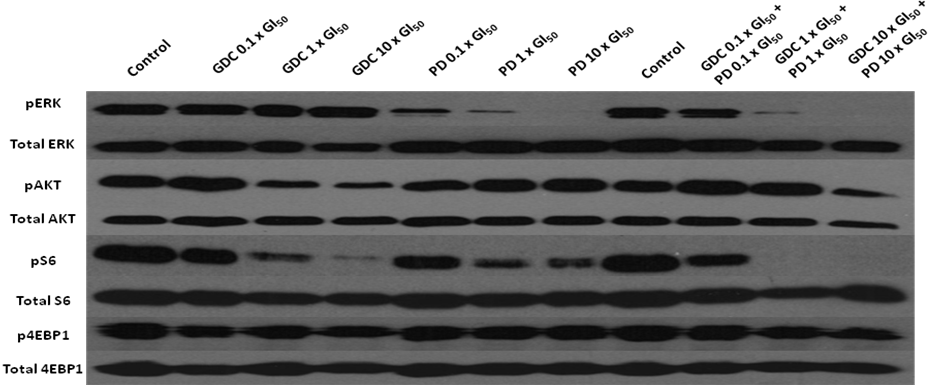


**HT29**


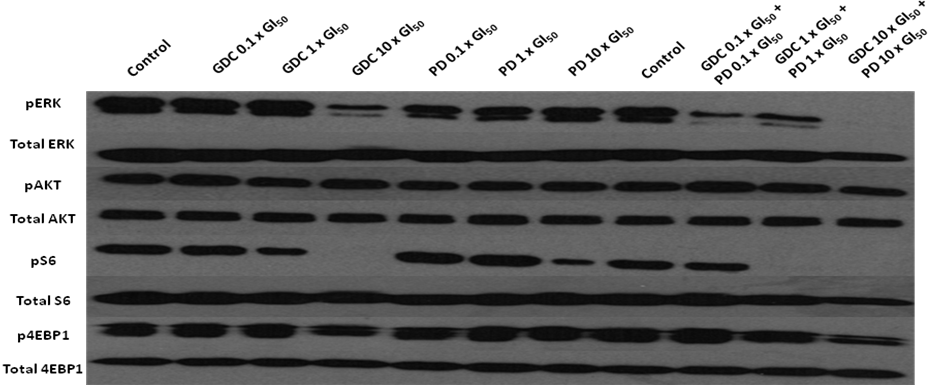


**Supplementary Figure S1**

Growth inhibition by the PI3K inhibitors NVP-BEZ235 and GDC-0941, the MEK inhibitors AZD6244 and PD0325901, and the mTOR inhibitor KU0063794. HCT116, DLD1 and HT29 cells were treated with the indicated concentrations of the inhibitors for 72 hours, and an SRB assay was subsequently performed. Data are presented as a percentage of the control, in which cells were treated with 0.5% (v/v) DMSO. Points represent the mean of ≥3 independent experiments ± standard error. Results were used to calculate the GI_50_ for each inhibitor and these values ± standard error are displayed below each graph.Lines were fitted using non-linear regression analysis.

**Supplementary Figure 2**

Effect of the PI3K/mTOR inhibitor NVP-BEZ235 or the PI3K inhibitor GDC-0941in combination with the MEK inhibitor, PD0325901, in DLD1 cells. **A.**Growth inhibition.DLD1 cells were treated with the indicated fractions of the GI_50_ concentration of the inhibitors (calculated from Supplementary Figure S1), alone or in combination for 72 hours, and an SRB assay was subsequently performed. Data are presented as a percentage of the control, in which cells were treated with 0.5% (v/v) DMSO. Points represent the mean of 3 independent experiments ± standard error and lines were fitted using non-linear regression analysis. **B.**Interaction of the combinations. Median effect analysis (CalcuSyn software) was used to evaluate the interaction between the inhibitor combinations. Points represent the mean of 3 independent experiments ± standard error. Horizontal dotted lines indicate the boundaries for each interaction classification.

**Supplementary Figure S3**

Cell survival after 72 hours exposure to the PI3K inhibitors NVP-BEZ235 and GDC-0941, and the MEK inhibitors AZD6244 and PD0325901. HCT116 and HT29 cells were treated with the indicated concentrations of the inhibitors for 72 hours, and cell survival was subsequently determined by clonogenic assay after 10-14 days of colony growth. Data are presented as a percentage of the control, in which cells were treated with 0.5% (v/v) DMSO. Points represent the mean of 3 independent replicates ± standard error. Lines were fitted using non-linear regression analysis.

**Supplementary Figure S4**

Cell survival after 72 hours exposure to the PI3K inhibitors NVP-BEZ235 (BEZ) or GDC-0941 (GDC), and the MEK inhibitors AZD6244 (AZD) or PD0325901 (PD), alone and in combination. HCT116 and HT29 cells were treated with a fixed concentration of each inhibitor alone or in combination for 72 hours, and cell survival was subsequently determined by clonogenic assay after 10-14 days of colony growth. Data are presented as a percentage of the control, in which cells were treated with 0.5% (v/v) DMSO. Bars represent the mean of 3 independent replicates ± standard error. * - Significantly different from either agent alone, p ≤ 0.05.

**Supplementary Figure S5**

Effect of the PI3K inhibitors NVP-BEZ235 (BEZ) or GDC-0941 (GDC), and the MEK inhibitor PD0325901 (PD), as single agents and in combination, on PI3K/AKT and MAPK signal transduction. HCT116 and HT29 cells were treated with the indicated concentrations of the inhibitors, derived from Supplementary Figure S1, alone or in combination, for 24 hours. Cell lysates were subjected to electrophoresis, followed by western blotting using the indicated phospho-specific antibodies. Blots were then stripped and re-probed with the corresponding total antibody to confirm equal protein loading. Data shown are representative of ≥3 independent experiments.

**Supplementary Figure S6**

Interaction of the PI3K inhibitors NVP-BEZ235 or GDC-0941, or the MEK inhibitors PD0325901 or AZD6244, in combination with the mTORC1/2 inhibitor KU0063794. Median effect analysis (CalcuSyn software) was used to evaluate the interaction between the inhibitor combinations. Points represent the mean of 3 independent experiments ± standard error. Horizontal dotted lines indicate the boundaries for each interaction classification.

**Supplementary Figure S7**Effect of the PI3K/mTOR inhibitor NVP-BEZ235 (BEZ) as a single agent as well as the PI3K inhibitor GDC-0941 (GDC) and the mTOR inhibitor KU0063794 (KU), as single agents and in combination, on PI3K/mTOR signal transduction. HCT116, DLD1 and HT29 cells were treated with the indicated concentrations of the inhibitors alone or in combination for 24 hours. Cell lysates were subjected to electrophoresis, followed by western blotting using the indicated phospho-specific antibodies. Blots were then stripped and re-probed with the corresponding total antibody to confirm equal protein loading. Data shown are representative of ≥3 independent experiments.

**NVP-BEZ235 and AZD6244 Combination**

|  | **Drug Concentration (Fraction of GI_50_)** | **Fraction affected by dose** | **Combination Index (CI)** | **Synergism/Antagonism** | |
| --- | --- | --- | --- | --- | --- |
| **HCT116** | 0.25 | 0.423 | 1.285 | -- | Moderate Antagonism |
|  | 0.5 | 0.642 | 0.560 | +++ | Synergism |
|  | 1 | 0.828 | 0.308 | +++ | Synergism |
|  | 2 | 0.932 | 0.215 | ++++ | Strong Synergism |
|  | 4 | 0.987 | 0.098 | +++++ | Very Strong Synergism |
| **HT29** | 0.25 | 0.438 | 0.839 | ++ | Moderate synergism |
|  | 0.5 | 0.706 | 0.544 | +++ | Synergism |
|  | 1 | 0.889 | 0.341 | +++ | Synergism |
|  | 2 | 0.976 | 0.237 | ++++ | Strong synergism |
|  | 4 | >0.999 | <0.070 | +++++ | Very strong synergism |

**GDC-0941 and AZD6244 Combination**

|  | **Drug Concentration (Fraction of GI_50_)** | **Fraction affected by dose** | **Combination Index (CI)** | **Synergism/Antagonism** | |
| --- | --- | --- | --- | --- | --- |
| **HCT116** | 0.25 | 0.680 | 0.259 | ++++ | Strong Synergism |
|  | 0.5 | 0.853 | 0.124 | ++++ | Strong Synergism |
|  | 1 | 0.962 | 0.039 | +++++ | Very Strong Synergism |
|  | 2 | >0.999 | <0.001 | +++++ | Very Strong Synergism |
|  | 4 | >0.999 | <0.002 | +++++ | Very Strong Synergism |
| **HT29** | 0.25 | 0.539 | 0.495 | +++ | Synergism |
|  | 0.5 | 0.710 | 0.361 | +++ | Synergism |
|  | 1 | 0.924 | 0.138 | ++++ | Strong synergism |
|  | 2 | >0.999 | <0.074 | +++++ | Very strong synergism |
|  | 4 | >0.999 | <0.057 | +++++ | Very strong synergism |

**NVP-BEZ235 and PD 0325901 Combination**

|  | **Drug Concentration (Fraction of GI_50_)** | **Fraction affected by dose** | **Combination Index (CI)** | **Synergism/Antagonism** | |
| --- | --- | --- | --- | --- | --- |
| **HCT116** | 0.25 | 0.411 | 0.792 | ++ | Moderate Synergism |
|  | 0.5 | 0.570 | 0.662 | +++ | Synergism |
|  | 1 | 0.796 | 0.376 | +++ | Synergism |
|  | 2 | 0.927 | 0.236 | ++++ | Strong Synergism |
|  | 4 | 0.980 | 0.144 | ++++ | Strong Synergism |
| **HT29** | 0.25 | 0.559 | 1.070 | ± | Nearly additive |
|  | 0.5 | 0.720 | 0.755 | ++ | Moderate synergism |
|  | 1 | 0.936 | 0.441 | +++ | Synergism |
|  | 2 | >0.999 | 0.241 | ++++ | Strong synergism |
|  | 4 | >0.999 | <0.111 | ++++ | Strong synergism |

**GDC-0941 and PD 0325901 Combination**

|  | **Drug Concentration (Fraction of GI_50_)** | **Fraction affected by dose** | **Combination Index (CI)** | **Synergism/Antagonism** | |
| --- | --- | --- | --- | --- | --- |
| **HCT116** | 0.25 | 0.653 | 0.268 | ++++ | Strong Synergism |
|  | 0.5 | 0.832 | 0.151 | ++++ | Strong Synergism |
|  | 1 | 0.959 | 0.045 | +++++ | Very Strong Synergism |
|  | 2 | >0.999 | <0.001 | +++++ | Very Strong Synergism |
|  | 4 | >0.999 | <0.002 | +++++ | Very Strong Synergism |
| **HT29** | 0.25 | 0.539 | 0.786 | ++ | Moderate synergism |
|  | 0.5 | 0.821 | 0.386 | +++ | Synergism |
|  | 1 | 0.968 | 0.097 | +++++ | Very strong synergism |
|  | 2 | >0.999 | <0.082 | +++++ | Very strong synergism |
|  | 4 | >0.999 | <0.032 | +++++ | Very strong synergism |

**Supplementary Table S1**

Interaction of PI3K and MEK inhibitor combinations. Median effect analysis (CalcuSyn software) was used to evaluate the interaction between the inhibitor combinations in Figure 1, and the data plotted graphically in Figure 2. Values represent the mean of 3 independent experiments.

|  | **Drug Concentration (Fraction of GI_50_)** | **Fraction affected by dose** | **Combination Index (CI)** | **Synergism/Antagonism** | |
| --- | --- | --- | --- | --- | --- |
| **BEZ/PD** | 0.25 | 0.6884 | 0.6180 | +++ | Synergism |
|  | 0.5 | 0.8738 | 0.2923 | ++++ | Strong synergism |
|  | 1 | 0.9762 | 0.0783 | +++++ | Very strong synergism |
|  | 2 | 1.0192 | 0.0010 | +++++ | Very strong synergism |
|  | 4 | 1.0247 | 0.0000 | +++++ | Very strong synergism |
| **GDC/PD** | 0.25 | 0.9120 | 0.0773 | +++++ | Very strong synergism |
|  | 0.5 | 0.9894 | 0.0130 | +++++ | Very strong synergism |
|  | 1 | 1.0186 | 0.0027 | +++++ | Very strong synergism |
|  | 2 | 1.0420 | 0.0000 | +++++ | Very strong synergism |
|  | 4 | 1.0433 | 0.0000 | +++++ | Very strong synergism |

**Supplementary Table S2**

Interaction of PI3K and MEK inhibitor combinations in the DLD1 cell line. Median effect analysis (CalcuSyn software) was used to evaluate the interaction between the inhibitor combinations in Supplementary Figure S2. Values represent the mean of 3 independent experiments.

**NVP-BEZ235 and KU0063794 Combination**

|  | **Drug Concentration (Fraction of GI_50_)** | **Fraction affected by dose** | **Combination Index (CI)** | **Synergism/Antagonism** | |
| --- | --- | --- | --- | --- | --- |
| **HCT116** | 0.25 | 0.7370 | 1.0787 | ± | Nearly additive |
|  | 0.5 | 0.5500 | 1.0573 | ± | Nearly additive |
|  | 1 | 0.3099 | 0.9467 | ± | Nearly additive |
|  | 2 | 0.1984 | 1.1813 | - | Slight antagonism |
|  | 4 | 0.1289 | 1.5983 | --- | Antagonism |
| **HT29** | 0.25 | 0.6788 | 0.8973 | + | Slight synergism |
|  | 0.5 | 0.5419 | 1.0697 | ± | Nearly additive |
|  | 1 | 0.3407 | 1.0520 | ± | Nearly additive |
|  | 2 | 0.2142 | 1.2213 | -- | Moderate antagonism |
|  | 4 | 0.1191 | 1.3540 | -- | Moderate antagonism |

**GDC-0941 and KU0063794 Combination**

|  | **Drug Concentration (Fraction of GI_50_)** | **Fraction affected by dose** | **Combination Index (CI)** | **Synergism/Antagonism** | |
| --- | --- | --- | --- | --- | --- |
| **HCT116** | 0.25 | 0.5194 | 0.6513 | +++ | Synergism |
|  | 0.5 | 0.3103 | 0.6743 | +++ | Synergism |
|  | 1 | 0.1277 | 0.5867 | +++ | Synergism |
|  | 2 | 0.0471 | 0.2353 | ++++ | Strong synergism |
|  | 4 | -0.0108 | 0.1973 | ++++ | Strong synergism |
| **HT29** | 0.25 | 0.6405 | 0.9317 | ± | Nearly additive |
|  | 0.5 | 0.4499 | 0.7500 | ++ | Moderate synergism |
|  | 1 | 0.2676 | 0.6007 | +++ | Synergism |
|  | 2 | 0.1208 | 0.4173 | +++ | Synergism |
|  | 4 | 0.0319 | 0.1840 | ++++ | Strong synergism |

**PD 0325901 and KU0063794 Combination**

|  | **Drug Concentration (Fraction of GI_50_)** | **Fraction affected by dose** | **Combination Index (CI)** | **Synergism/Antagonism** | |
| --- | --- | --- | --- | --- | --- |
| **HCT116** | 0.25 | 0.5628 | 0.6093 | +++ | Synergism |
|  | 0.5 | 0.3919 | 0.4837 | +++ | Synergism |
|  | 1 | 0.2425 | 0.3937 | +++ | Synergism |
|  | 2 | 0.1240 | 0.2987 | ++++ | Strong synergism |
|  | 4 | 0.0516 | 0.2163 | ++++ | Strong synergism |
| **HT29** | 0.25 | 0.4975 | 0.7957 | ++ | Moderate synergism |
|  | 0.5 | 0.2849 | 0.5993 | +++ | Synergism |
|  | 1 | 0.1355 | 0.4377 | +++ | Synergism |
|  | 2 | 0.0438 | 0.2223 | ++++ | Strong synergism |
|  | 4 | -0.0104 | 0.0880 | +++++ | Very strong synergism |

**AZD6244 and KU0063794 Combination**

|  | **Drug Concentration (Fraction of GI_50_)** | **Fraction affected by dose** | **Combination Index (CI)** | **Synergism/Antagonism** | |
| --- | --- | --- | --- | --- | --- |
| **HCT116** | 0.25 | 0.4829 | 0.4610 | +++ | Synergism |
|  | 0.5 | 0.3300 | 0.3000 | +++ | Synergism |
|  | 1 | 0.1903 | 0.2243 | ++++ | Strong synergism |
|  | 2 | 0.0769 | 0.1407 | ++++ | Strong synergism |
|  | 4 | 0.0008 | 0.0590 | +++++ | Very strong synergism |
| **HT29** | 0.25 | 0.6445 | 0.8507 | + | Slight synergism |
|  | 0.5 | 0.4489 | 0.7203 | ++ | Moderate synergism |
|  | 1 | 0.2552 | 0.5737 | +++ | Synergism |
|  | 2 | 0.1151 | 0.4193 | +++ | Synergism |
|  | 4 | 0.0288 | 0.1903 | ++++ | Strong synergism |

**Supplementary Table S3**

Interaction of the PI3K inhibitors NVP-BEZ235 or GDC-0941, or the MEK inhibitors PD0325901 or AZD6244, in combination with the mTORC1/2 inhibitor KU0063794. Median effect analysis (CalcuSyn software) was used to evaluate the interaction between the inhibitor combinations. The data are plotted graphically in Supplementary Figure S6, and values represent the mean of 3 independent experiments.

**NVP-BEZ235 andKU 0063794 in combination with PD 0325901**

|  | **Drug Concentration (Fraction of GI_50_)** | **Fraction affected by dose** | **Combination Index (CI)** | **Synergism/Antagonism** | |
| --- | --- | --- | --- | --- | --- |
| **HCT116** | 0.25 | 0.5019 | 1.0897 | ± | Nearly additive |
|  | 0.5 | 0.6930 | 0.5767 | +++ | Synergism |
|  | 1 | 0.8658 | 0.2880 | ++++ | Strong synergism |
|  | 2 | 0.9572 | 0.1380 | ++++ | Strong synergism |
|  | 4 | 1.0161 | 0.0090 | +++++ | Very strong synergism |
| **HT29** | 0.25 | 0.4762 | 0.6940 | +++ | Synergism |
|  | 0.5 | 0.6287 | 0.7463 | ++ | Moderate synergism |
|  | 1 | 0.8110 | 0.5760 | +++ | Synergism |
|  | 2 | 0.9100 | 0.5020 | +++ | Synergism |
|  | 4 | 0.9635 | 0.4100 | +++ | Synergism |

**GDC-0941 and KU 0063794 in combination with PD 0325901**

|  | **Drug Concentration (Fraction of GI_50_)** | **Fraction affected by dose** | **Combination Index (CI)** | **Synergism/Antagonism** | |
| --- | --- | --- | --- | --- | --- |
| **HCT116** | 0.25 | 0.6097 | 0.5250 | +++ | Synergism |
|  | 0.5 | 0.7834 | 0.3067 | +++ | Synergism |
|  | 1 | 0.9366 | 0.1053 | ++++ | Strong synergism |
|  | 2 | 0.9900 | 0.0267 | +++++ | Very strong synergism |
|  | 4 | 1.0210 | 0.0050 | +++++ | Very strong synergism |
| **HT29** | 0.25 | 0.4998 | 0.6583 | +++ | Synergism |
|  | 0.5 | 0.7233 | 0.4663 | +++ | Synergism |
|  | 1 | 0.8740 | 0.3310 | +++ | Synergism |
|  | 2 | 0.9478 | 0.2413 | ++++ | Strong synergism |
|  | 4 | 0.9950 | 0.0800 | +++++ | Very strong synergism |

**Supplementary Table S4**

Interaction of the PI3K/mTOR inhibitor NVP-BEZ235, or the PI3K inhibitor GDC-0941, mixed at equipotent concentrations with the mTORC1/2 inhibitor, KU0063794, in combination with the MEK inhibitor PD0325901. Median effect analysis (CalcuSyn software) was used to evaluate the interaction between the inhibitor combinations. The data are plotted graphically in Figure 4, and values represent the mean of 3 independent experiments.

| **Inhibitor combined with PD 0325901** | **Average Combination Index at 1 x GI_50_** | **Synergism/Antagonism** | |
| --- | --- | --- | --- |
| NVP-BEZ235 | 0.376 | +++ | Synergism |
| KU0063794 | 0.460 | +++ | Synergism |
| 0.01 GDC: 1 KU | 0.343 | +++ | Synergism |
| 1 GDC: 1 KU | 0.231 | ++++ | Strong synergism |
| 100 GDC: 1 KU | 0.080 | +++++ | Very strong synergism |
| 10000 GDC: 1 KU | 0.086 | +++++ | Very strong synergism |
| GDC-0941 | 0.084 | +++++ | Very strong synergism |

**Supplementary Table S5**

Relationship between the molar ratio of the PI3K inhibitor GDC-0941, to the mTORC1/2 inhibitor KU0063794, and synergy with the MEK inhibitor PD0325901 in the HCT116 cell line. HCT116 cells were treated with the PI3K inhibitor GDC-0941, the mTORC1/2 inhibitor KU0063794, or the indicated molar ratios of the two inhibitors, in combination with the MEK inhibitor PD0325901 for 72 hours, and an SRB assay was subsequently performed. Median effect analysis (CalcuSyn software) was used to evaluate the interaction between the inhibitor combinations. Values represent the mean of the combination indices at the GI_50_ concentration for 3 independent experiments.

| **Inhibitor combined with PD 0325901** | **Average Combination Index at 1 x GI_50_** | **Synergism/Antagonism** | |
| --- | --- | --- | --- |
| NVP-BEZ235 | 0.292 | ++++ | Strong synergism |
| KU0063794 | 0.277 | ++++ | Strong synergism |
| 0.01 GDC: 1 KU | 0.259 | ++++ | Strong synergism |
| 1 GDC: 1 KU | 0.123 | ++++ | Strong synergism |
| 100 GDC: 1 KU | 0.022 | +++++ | Very strong synergism |
| 10000 GDC: 1 KU | 0.034 | +++++ | Very strong synergism |
| GDC-0941 | 0.013 | +++++ | Very strong synergism |

**Supplementary Table S6**

Relationship between the molar ratio of the PI3K inhibitor GDC-0941, to the mTORC1/2 inhibitor KU0063794, and synergy with the MEK inhibitor PD0325901 in the DLD1 cell line. DLD1 cells were treated with the PI3K inhibitor GDC-0941, the mTORC1/2 inhibitor KU0063794, or the indicated molar ratios of the two inhibitors, in combination with the MEK inhibitor PD0325901 for 72 hours, and an SRB assay was subsequently performed. Median effect analysis (CalcuSyn software) was used to evaluate the interaction between the inhibitor combinations. Values represent the mean of the combination indices at 0.5x the GI_50_ concentration for 3 independent experiments.

| **Inhibitor combined with PD 0325901** | **Average Combination Index at 1 x GI_50_** | **Synergism/Antagonism** | |
| --- | --- | --- | --- |
| NVP-BEZ235 | 0.441 | +++ | Synergism |
| KU0063794 | 0.551 | +++ | Synergism |
| 0.01 GDC: 1 KU | 0.670 | +++ | Synergism |
| 1 GDC: 1 KU | 0.353 | +++ | Synergism |
| 100 GDC: 1 KU | 0.420 | +++ | Synergism |
| 10000 GDC: 1 KU | 0.228 | ++++ | Strong synergism |
| GDC-0941 | 0.000 | +++++ | Very strong synergism |

**Supplementary Table S7**

Relationship between the molar ratio of the PI3K inhibitor GDC-0941, to the mTORC1/2 inhibitor KU0063794, and synergy with the MEK inhibitor PD0325901 in the HT29 cell line. HT29 cells were treated with the PI3K inhibitor GDC-0941, the mTORC1/2 inhibitor KU0063794, or the indicated molar ratios of the two inhibitors, in combination with the MEK inhibitor, PD0325901, for 72 hours, and an SRB assay was subsequently performed. Median effect analysis (CalcuSyn software) was used to evaluate the interaction between the inhibitor combinations. Values represent the mean of the combination indices at the GI_50_ concentration for 3 independent experiments.

**Supplementary materials and methods**

**Growth Inhibition Assay**

Exponentially growing cells were exposed to increasing concentrations of the single agent inhibitors, or of NVP-BEZ235 or GDC-0941 combined with AZD6244 or PD0325901, KU0063794 combined with each of the 4 inhibitors, or molar ratios of GDC-0941:KU0063794 combined with PD 0325901 at 0.25, 0.5, 1, 2 or 4 times their half maximal growth inhibitory concentration (GI_50_) in DMSO, or 0.5% DMSO alone, for 72 hours before fixing in 50% (w/v) trichloroacetic acid (TCA) and staining with 0.4% (w/v) SRB in 1% (v/v) acetic acid. The stain was then solubilised with 10 mM Tris pH 10.5 and the absorbance was read at 570 nm using a SpectroMax 250 microplate spectrophotometer system (Molecular Devices Corporation, Sunnyvale, California, USA). The GI_50_ concentration was calculated based on a standard point to point curve with 1000 segments using GraphPad Prism software (La Jolla, California, USA). The data were analysed by median effect analysis using CalcuSyn software (Biosoft, Great Shelford, Cambridgeshire, UK), which calculates the combination index of multiple drugs by an algebraic estimation algorithm.

**Cytotoxicity Assay**

Exponentially growing cells were exposed to increasing concentrations of the single agents or 10 µM of AZD6244 or PD0325901, combined with 0.1, 1 or 10 µM NVP-BEZ235 or 10 µM GDC-0941 in DMSO or 0.5% (v/v) DMSO alone for 72 hours before harvesting and reseeding for colony formation. After growth for 10-14 days, colonies were fixed in methanol–acetic acid 3:1 (v/v) and stained with crystal violet (0.4% w/v). Colonies consisting of more than 50 cells were counted on an automated colony counter (Oxford Optronix, Oxford, UK). Two-tailed paired t tests were used to compare the different groups. Differences with a p < 0.05 were considered statistically significant.

**Western Blotting**

Cells were treated with the inhibitors in DMSO alone or in combination at 0.1, 1 or 10 times their respective GI_50_ values for 24 hours, with the exception of KU0063794 which was used alone and in combination at the GI_50_ value determined for GDC-0941. Extracts were then prepared in PhosphoSafe™ extraction reagent (Merck Chemicals Ltd, Nottingham, Nottinghamshire, UK) and a protease inhibitor cocktail kit (Pierce, Thermo Scientific, Rockford, Illinois, USA). Proteins were resolved on Novex® 4-12% (w/w) Tris-glycine gels (Invitrogen Ltd, Renfrew, Paisley, UK) and electrotransferred onto Hybond C nitrocellulose membrane (GE Healthcare Life Sciences, Hatfield, Hertfordshire, UK). Membranes were incubated with phospho-4EBP1 (Thr37/46) (#2855), phospho-p44/42 MAPK (Thr202/Tyr204) (#4370), phospho-Akt (Ser473) (#4060) or phospho-S6 ribosomal protein (Ser235/236) (#4858) monoclonal antibodies obtained from Cell Signalling Technology (New England BioLabs (UK) Ltd, Hitchin, Hertfordshire, UK). Antibody binding was detected by incubation with a HRP-conjugated goat anti-rabbit polyclonal antibody (Dako, Glastrop, Denmark). Blots were developed using Pierce ECL (enhanced chemiluminescence) western blotting substrate (Thermo Scientific, Rockford, Illinois, USA), or SuperSignal® West Dura extended duration substrate (Thermo Scientific, Rockford, Illinois, USA), and Kodak X-ray film (Genetic Research Instrumentation Ltd, Braintree, Essex, UK) on MediPhot 937 film developer (Colenta, Weiner Neustadt, Austria), then digitally scanned.
