# Supplementary figures and images for: The synergistic interaction of MEK and PI3K inhibitors is modulated by mTOR inhibition
Source: Br J Cancer. 2012 Mar 13;106(8):1386–94. doi: 10.1038/bjc.2012.70 (PMC3326670; doi:10.1038/bjc.2012.70)

**Supplementary Figure S2**

**A**


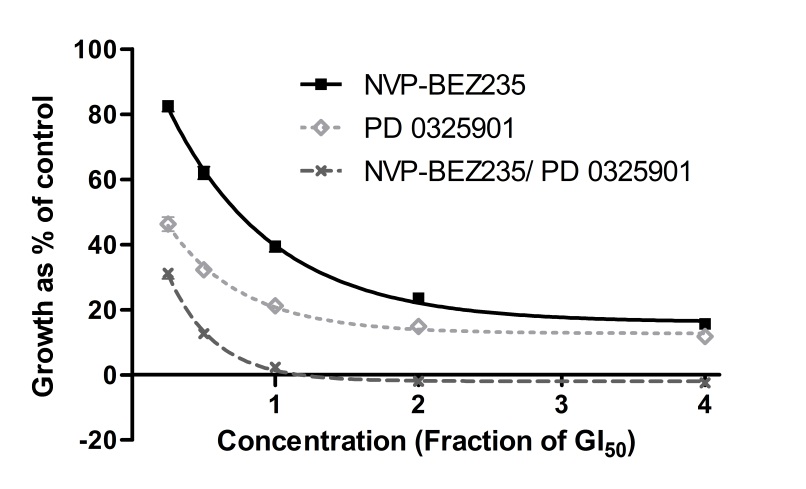


**B**


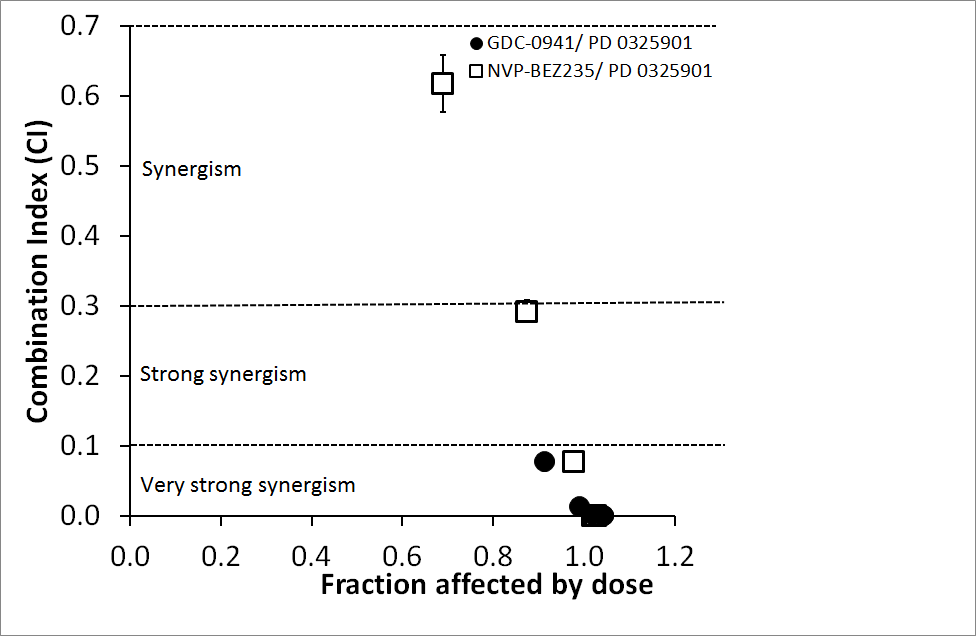


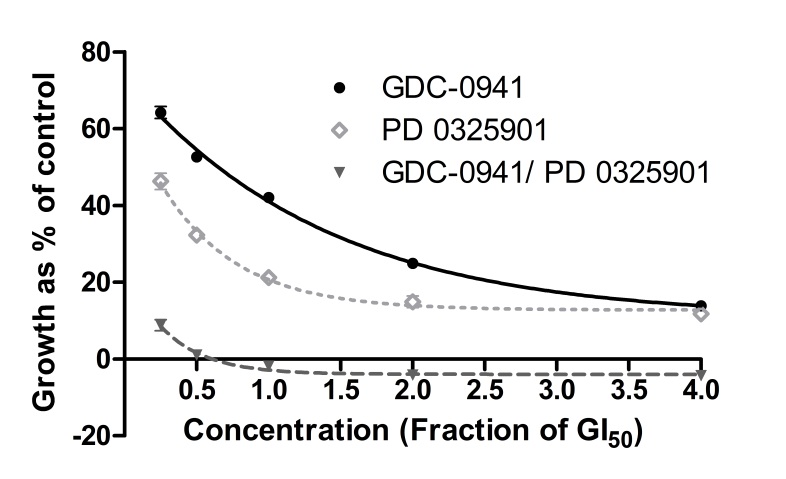

Supplement: Supplementary Figure 2 [file bjc201270x2.doc]

**Supplementary Figure S7**

**HCT116**

**
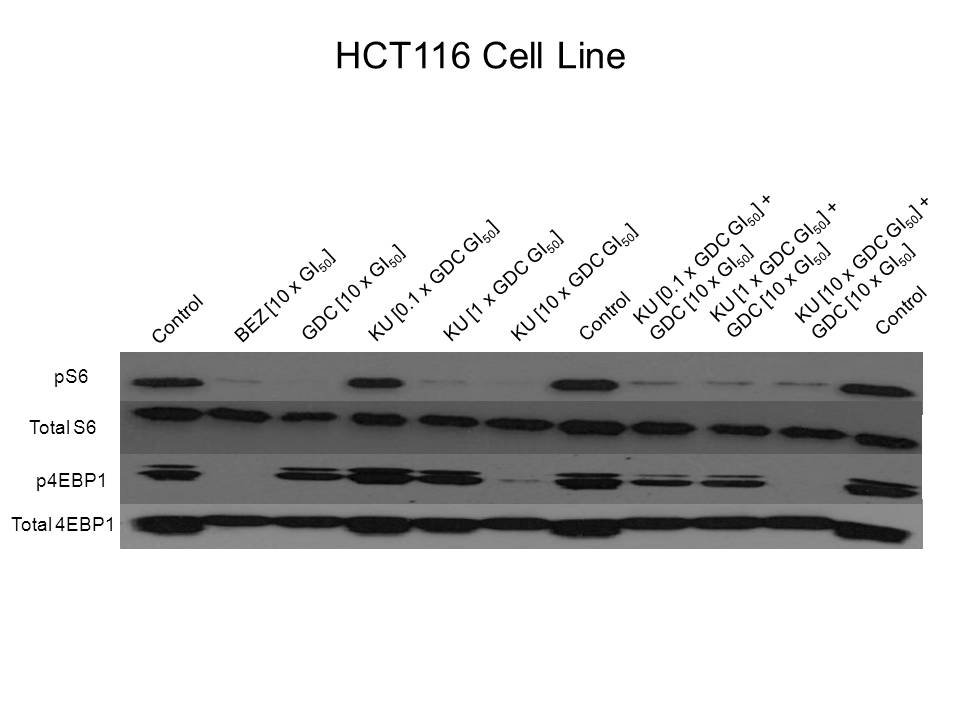
**

**DLD1**

**
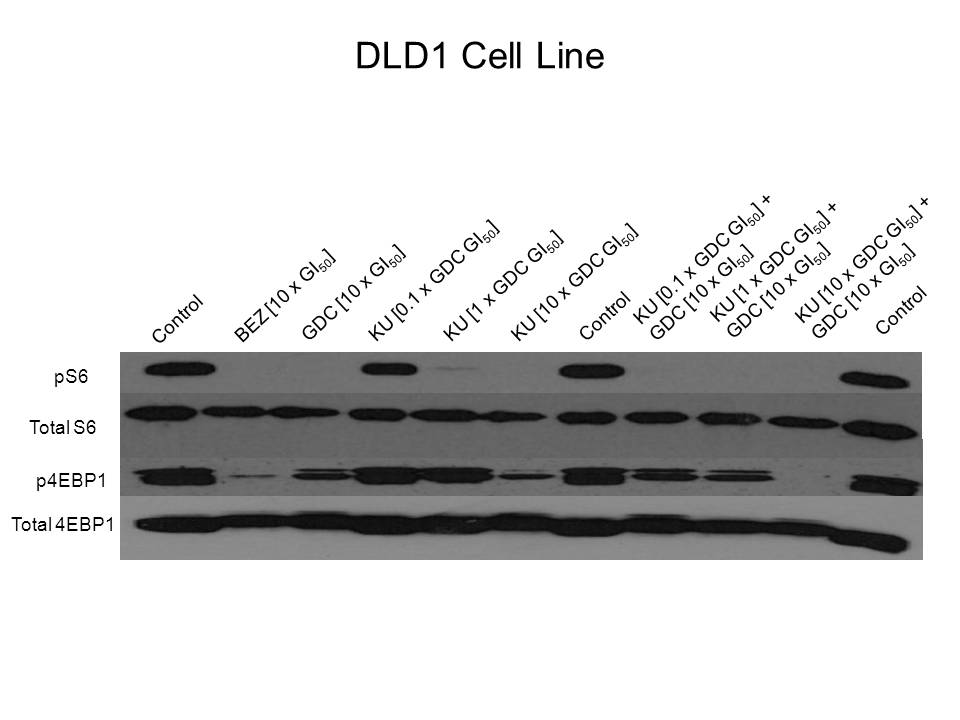
**

**HT29**

**
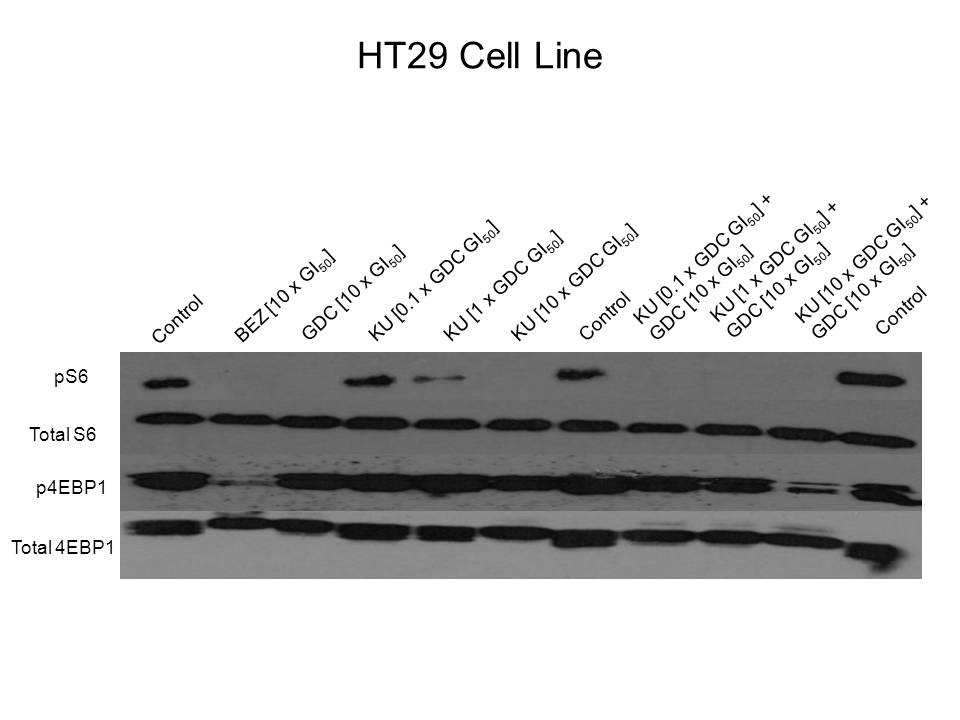
**

Supplement: Supplementary Figure 7 [file bjc201270x3.doc]
